# Neuromorphic spike-based large language model

**DOI:** 10.1093/nsr/nwaf551

**Published:** 2025-12-04

**Authors:** Han Xu, Xuerui Qiu, Yunhui Xu, Mohammed E Elbtity, Peng Zhou, Yang Tian, Rui-Jie Zhu, Jiahong Zhang, Shaowei Gu, Yuqi Pan, Yuhong Chou, Qinghao Wen, Man Yao, Jiangbo Qian, Yonghong Tian, Lei Ma, Tiejun Huang, Jason K Eshraghian, Bo Xu, Guoqi Li

**Affiliations:** Institute of Automation, Chinese Academy of Sciences, Beijing 100190, China; School of Artificial Intelligence, University of Chinese Academy of Sciences, Beijing 100049, China; Brain-Inspired Models Group, Beijing Academy of Artificial Intelligence, Beijing 100862, China; Institute of Automation, Chinese Academy of Sciences, Beijing 100190, China; School of Future Technology, University of Chinese Academy of Sciences, Beijing 101408, China; Zhongguancun Academy, Beijing 100094, China; Department of Psychology, Tsinghua University, Beijing 100084, China; Advanced Micro Devices Inc, Santa Clara 95054, USA; Department of Research, LuxiTech, Shenzhen 518055, China; Faculty of Data Science, City University of Macau, Macau 999078, China; Department of Electrical and Computer Engineering, University of California, Santa Cruz 95064, USA; Institute of Automation, Chinese Academy of Sciences, Beijing 100190, China; School of Artificial Intelligence, University of Chinese Academy of Sciences, Beijing 100049, China; Institute of Automation, Chinese Academy of Sciences, Beijing 100190, China; School of Future Technology, University of Chinese Academy of Sciences, Beijing 101408, China; Zhongguancun Academy, Beijing 100094, China; Institute of Automation, Chinese Academy of Sciences, Beijing 100190, China; Department of Data Science and Artificial Intelligence, The Hong Kong Polytechnic University, Hong Kong 999077, China; Institute of Automation, Chinese Academy of Sciences, Beijing 100190, China; School of Aerospace, Mechanical and Mechatronic Engineering, The University of Sydney, Sydney 2006, Australia; Institute of Automation, Chinese Academy of Sciences, Beijing 100190, China; Faculty of Electrical Engineering and Computer Science, Ningbo University, Ningbo 315211, China; School of Computer Science, Peking University, Beijing 100871, China; Brain-Inspired Models Group, Beijing Academy of Artificial Intelligence, Beijing 100862, China; School of Computer Science, Peking University, Beijing 100871, China; Brain-Inspired Models Group, Beijing Academy of Artificial Intelligence, Beijing 100862, China; School of Computer Science, Peking University, Beijing 100871, China; Department of Electrical and Computer Engineering, University of California, Santa Cruz 95064, USA; Institute of Automation, Chinese Academy of Sciences, Beijing 100190, China; School of Artificial Intelligence, University of Chinese Academy of Sciences, Beijing 100049, China; Institute of Automation, Chinese Academy of Sciences, Beijing 100190, China; School of Artificial Intelligence, University of Chinese Academy of Sciences, Beijing 100049, China; Key Laboratory of Brain Cognition and Brain-inspired Intelligence Technology, Beijing 100190, China; Spiking Intelligence Lab, Tianqiao & Chirssy Chen Institute, Shanghai 201203, China

**Keywords:** neuromorphic computing, spike-based LLM, spiking linear attention, interdisciplinary neuroscience

## Abstract

This work proposes a unified neuromorphic spike-based large-language-model (NSLLM) framework to simultaneously address the challenges of high energy consumption and low interpretability in LLMs. Our framework transforms LLMs into efficient NSLLMs by converting their behaviors into neural dynamics—such as spike trains—through rigorous mathematical modeling and complemented by advanced techniques including quantization and sparsification. This transformation also enables the analysis of information encoding processes using computational neuroscience tools, thereby offering a novel neuroscientific perspective that conceptualizes LLMs as neural populations to enhance their interpretability. Leveraging a hardware-algorithm co-design paradigm, an NSLLM can completely eliminate matrix multiplication (MatMul) while maintaining high performance. We designed a custom MatMul-free hardware core on the VCK190 field-programmable gate array to validate the 1.5-billion-parameter NSLLM, achieving a dynamic power consumption of only 13.849 W and an inference throughput of 161.8 tokens per second. Compared with the A800 GPU, this implementation improves energy efficiency, memory usage and inference throughput by 19.8$\times$, 21.3$\times$ and 2.2$\times$, respectively. This work provides a novel perspective within a unified framework to enhance both the energy efficiency and interpretability of LLMs, offering valuable insights for future neuromorphic chip designs tailored for large models.

## Teaser

This work proposes a unified neuromorphic spike-based LLMs (NSLLM) framework to simultaneously address the challenges of high energy consumption and low interpretability in LLMs.

## INTRODUCTION

Large language models (LLMs) have emerged as a dominant paradigm in the pursuit of artificial general intelligence (AGI) [1,2], yet they still face several limitations. First, these methods come with substantial computational and memory implementation costs [3,4]. In contrast to the one-time cost associated with model training, the inference process of LLMs is repeatedly executed across diverse users. The huge resource cost poses a significant constraint on their potential to serve as a foundational infrastructure for human society. Second, current LLMs lack interpretability from the perspective of biological intelligent systems [5,6]. This not only hinders the long-term development planning of AI systems [7], but also restricts their transparency and reliability in critical applications, e.g. the decision-making and optimization processes in high-stakes healthcare and finance fields. In contrast, the human brain performs complex tasks including language processing and decision-making with remarkable efficiency, consuming less than 20 W of energy and operating through a multi-scale functional organization [8,9]. This stark contrast underscores the gap between LLMs and human cognition, highlighting dual challenges. On the one hand, there is an urgent demand for solutions

that can enhance the computational efficiency of LLMs to boost energy efficiency and economize on human resources. On the other hand, it is essential to improve the interpretability [10,11] of mechanisms within these models, thereby deepening our comprehension of the interactions and functions of components within large-scale systems.

However, existing research typically addresses these two challenges separately and lacks a systematic methodology to solve this fundamental issue. The bottleneck lies in the scarcity of technical development in low-power large-model technologies and analytical methodologies for interpretability, particularly those based on interdisciplinary studies. For instance, commonly used quantization methods enhance energy efficiency by reducing the bit-width of model parameters and activations, albeit at the cost of performance degradation [12,13]; efforts to improve interpretability predominantly focus on elucidating the internal mechanisms of neural networks through statistical methods [14,15], visualization techniques [16,17], gradient analysis [18,19] and related approaches. By integrating insights from neuroscience and computational science, this work proposes a unified neuromorphic, spike-based large-language-model (NSLLM) architecture—designed to emulate the structure and functionality of the brain—which enhances their energy efficiency while simultaneously introducing interpretability.

To address energy-efficiency challenges, we adopt an algorithm-hardware co-design approach to construct an NSLLM at the billion-parameter scale. Leveraging its sparse, spike-driven characteristics, we design a matrix-multiplication-free (MatMul-free) hardware architecture, which is deployed and validated on a field-programmable gate array (FPGA) platform. During the training stage on a GPU, the continuous outputs of LLMs are converted into discrete integer spikes, as in traditional activation-value quantization methods. A combined strategy of layer-wise directed sparse quantization and mixed-precision training is proposed to maintain lower inference spike-firing rates while reducing computational costs, thereby balancing model performance under low-bit activations, supporting models up to 13B parameters. We designed a ternary hardware core on the VCK190 FPGA [20], which efficiently transforms multiplication operations into addition, subtraction or skip operations, to compute multi-step binary spike trains generated from integer spikes, thereby completely eliminating the reliance on computationally expensive MatMul. As a result of these optimizations, our NSLLM achieves remarkable efficiency metrics. Specifically, the power consumption is reduced to only 13.849 W, while maintaining a throughput of 161.8 tokens per second. Importantly, these gains are achieved while maintaining competitive performance, even at the billion-parameter scale. Compared to running conventional LLMs [21–23] on the A800 GPU, our approach demonstrates a 19.8$\times$ improvement in power efficiency, a 21.3$\times$ reduction in memory usage and a 2.2$\times$ increase in inference throughput.

To understand an NSLLM as an interpretability framework, since we have converted LLMs into neuromorphic models by transforming the behaviors of LLMs into multi-step binary spike trains, we can model the spiking neural populations through a mathematically rigorous process. The spatiotemporal spike trains can be regarded as outcomes of the probabilistic firing models with our analytical framework, which enables us to evaluate the temporal dynamics of neurons based on the probability distribution of spiking activity derived from the spike trains. Then, LLMs are modeled as spiking neural populations with an NSLLM, thereby enabling the measurement of key information-theoretic metrics—including Kolmogorov–Sinai entropy [24]), Shannon entropy and mutual information—during language processing [25], and offering interpretable insights while significantly reducing data requirements. Experimental results indicate that the model can distinguish between ambiguous and unambiguous texts by encoding information more effectively when processing unambiguous texts. This deeper understanding of LLMs as spiking neural populations both improves resource efficiency and enhances interpretability.

In summary, by drawing on insights from neuroscience, an NSLLM enables the transformation of any pre-trained LLM into an energy-efficient and interpretable model. Neuroscience indicates that biological systems optimize energy consumption through sparse and event-driven computation, and facilitate information flow through meaningful neuronal events, enhancing system interpretability. The proposed framework not only advances the state of the art in energy-efficient AI, but also offers a novel perspective on AGI development, where biological efficiency, interpretability and computational power converge to create more adaptable and sustainable intelligent systems.

## RESULTS

### Overview of the NSLLM

The NSLLM acts as a bridge between deep learning and neuroscience, constructing LLMs that are not only efficient but also interpretable, thus overcoming the scalability divide between these two fields. In sharp contrast to traditional LLMs, the NSLLM leverages low-power, biologically plausible spiking neural networks to construct expansive language models, seamlessly uniting interpretability and efficiency within a cohesive framework.

We introduce key components of the NSLLM, which employs single-time-step, multi-bit spike neurons in place of spike neural networks trained with back-propagation through time to compress spatiotemporal dynamics and reduce training overhead. During inference, extended channels generate binary spike trains with adjustable types, preserving neuromorphic fidelity. Our spike linear attention achieves linear time complexity and reduces computation by replacing matrix multiplications with sparse additions. To enhance energy efficiency, we apply layer-wise quantization guided by sensitivity metrics, alongside mixed-precision training and a sparsity method that modifies membrane potentials to increase zero activations and lower firing rates.

To validate the NSLLM’s energy efficiency, we deployed it on AMD’s Versal (VCK190) FPGA using its AI engine and programmable logic for a billion-parameter MatMul-free LLM. This design includes vector operation cores and a central CPU. By replacing matrix multiplications with additions, it maintains performance while significantly reducing energy use and improving resource utilization. Compared to conventional deployments, this approach offers a more cost-effective and energy-efficient solution for LLMs.

To demonstrate enhanced interpretability, we model the NSLLM as a spiking neural population and analyze its behavior using entropy-based metrics (e.g. Kolmogorov–Sinai entropy for neural dynamics [24], and Shannon entropy or mutual information for information processing [25]). Experiments show that the model encodes information more effectively for unambiguous texts, enabling it to differentiate text types. By combining neural dynamics with information-theoretic analysis, the framework yields biologically interpretable insights into LLM mechanisms with reduced data requirements.

In conclusion, we have released numerous pre-trained models with varying parameter scales (e.g. from 169 million to 13 billion parameters), different quantization bit-widths for integer spikes and weights, optional sparsity strategies and integrated mixed-precision training. Our evaluation experiments show that, compared with traditional LLMs, our approach achieves comparable performance while maintaining the energy efficiency of spike-based computation. This work, based on the concept of a unified network architecture with interchangeable neurons, successfully demonstrates the feasibility of building an equivalent conversion bridge between LLMs and spike-based LLMs. In the remaining subsections of the Results section, we discuss the integration of our spiking neurons with spiking linear attention. We then cover spiking quantization and spiking sparsification. This is followed by a multi-dimensional performance analysis of the NSLLM. Finally, we present the FPGA energy-efficiency and interpretability results in detail.

### NSLLM with flexibly adjustable integer spiking neurons to bridge deep learning and neuroscience

Inspired by the spike-driven communication mechanism of biological neurons, spiking neurons exhibit both spatial and temporal attributes. This characteristic confers on spiking neurons remarkable temporal-information-processing competencies, frequently leading to enhanced performance in specific tasks. However, spiking neurons depend on discrete spike trains to encode information, a reliance that intrinsically restricts their representational capacity. Additionally, the process of transforming membrane potentials into binary spikes through quantization gives rise to supplementary quantization errors, thereby imposing further constraints on the ability of model representation. Consequently, how to effectively utilize the advantages of spiking neurons in temporal information processing while simultaneously minimizing quantization errors has emerged as the most crucial issue.

**Figure 1.**
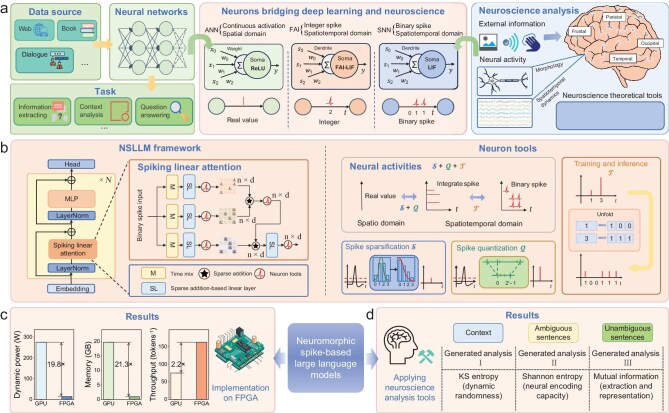
NSLLM: an efficiency processing framework from large language models to neuromorphic architectures. (a) NSLLM introduces FAI-LIF neurons to convert conventional LLMs into spike trains, supporting the analysis of spike signals and neural dynamics and bridging LLMs with neuromorphic systems. (b) The unified architecture uses spiking linear attention to convert softmax attention to linear complexity, where sparse additions replace matrix multiplication. Core neuron tools include spike sparsification ($\mathcal{S}$) and spike quantization ($\mathcal{Q}$), which map continuous neural activities to integer spikes and unfold them into sparse binary spike trains across virtual time steps during training and inference ($\mathcal{T}$). (c) At the billion-parameter scale, NSLLM's FPGA implementation enables MatMul-free inference while maintaining model performance, with a dynamic power of 13.849 W and improvements of 19.8×, 21.3×, and 2.2× in power efficiency, memory usage, and inference throughput over the A800 GPU. (d) NSLLM interpretability is analyzed by modeling it as a spiking neural population and examining ambiguous and unambiguous sentences using KS entropy, Shannon entropy, and mutual information to characterize dynamic randomness, encoding capacity, and information representation, yielding neuroscience-inspired insights.

To tackle this challenge, we have devised a model founded on flexibly adjustable integer spiking neurons (FAI-SN). As depicted in Fig. 1a, we meticulously monitored the quantization errors of spiking neurons and quantized the full-precision activation values and weights within predefined integer intervals (details can be found in the subsection entitled ‘Spiking neuron models’ in the Methods section below). Figure 1c (right) illustrates the entire process of integer spiking neurons from training to inference. During training, we integrated leaky integrate-and-analog-fire (LIAF) neurons [26] with traditional artificial neural network layers and used single-time-step integer spike-firing values to reduce the quantization errors of spiking neurons. This integration strategy not only enhances training performance, but also effectively reduces training costs. In the inference phase, we employed leaky integrate-and-fire (LIF) neurons [27] to convert integer spike-firing values into binary spikes over extended virtual time steps (details can be found in the subsection entitled ‘Spike neuron tools’ in the Methods section), preserving the temporal characteristics of spiking neural networks. Since the NSLLM employs binary pulses to transmit information, matrix multiplication operations can be simplified to sparse additions. This further reduces power consumption during inference when deployed on hardware.

In the NSLLM, we have provided flexible options for selecting the type of spike-firing values. As shown in Fig. 1a, in the extreme case, choosing binary spike trains can significantly reduce power consumption and facilitate hardware deployment. In contrast, using floating-point numbers can lead to better performance. There is a trade-off between these two approaches, and our neurons allow the output to switch between binary and floating-point values as needed. In practical applications, to optimize the model’s performance under different task requirements and computational resource constraints, we balance the trade-off between power efficiency and accuracy by dynamically selecting the type of spike trains. Additionally, the flexible selection brings a secondary yet significant benefit: it provides an opportunity to transform the LLMs into large-scale neural populations that can be analyzed using neuroscience tools. Although such analysis is not an essential part of our technical framework, it creates possibilities for studying the behavior of LLMs from a neuroscience perspective, allowing us to analyze the dynamic characteristics and informational functions of each neuron within LLMs. In the following sections, we demonstrate these possibilities to suggest the potential of our framework as a bridge between deep learning and neuroscience.

### NSLLM with a spiking linear attention mechanism to improve computational efficiency

The quadratic complexity of the self-attention [28] mechanism arises from the full pairwise computation of attention scores across all token positions. This results in a time complexity that grows proportionally to the square of the sequence length, which poses significant computational challenges when handling long sequence data, substantially increasing the computational cost of model training and inference. In contrast, linear attention mechanisms that adopt recurrent neural network (RNN)-style temporal dependencies reduce the time complexity to linear by considering each token’s dependency only on the previous time step. However, this temporal dependency limits the parallelization capability of linear attention models and constrains their ability to capture complex dependencies between tokens in a sequence [29,30].

In the NSLLM, we designed a spiking linear attention mechanism based on the relative position encoding and key-value separation technique of the RWKV [22] architecture, as shown in Fig. 1b (details can be found in the subsection entitled ‘Spike linear attention’ in the Methods section below). This mechanism eliminates the dependence on sequential computation, enabling efficient parallel training while capturing the relative relationships among elements in a sequence, which enhances the model’s ability to model long sequence data. Furthermore, the key-value separation technique avoids the explicit interaction between queries and keys in traditional attention mechanisms, reducing the complexity from $O(L^2 d)$ to $O(L d)$, where $L$ is the sequence length and $d$ is the feature dimension. This significantly optimizes the efficiency of attention weight computation and improves the model’s scalability.

Moreover, we integrated sparse spiking neurons into the spiking linear attention mechanism to further reduce computational complexity. In our design, we implemented a global regulation strategy for both activation values and weights. We deployed fake quantization modules in every network layer that could be converted to spiking neuron layers during inference. For network layers that demand normalization or accumulation operations on membrane potentials, including normalization layers, residual connections and time-shift modules used for encoding relative positions, we seamlessly incorporated these layers into the spiking neuron layers. When dealing with quantized integer spike values, we adopted the Rescale operation for arithmetic calculations. Specifically, the Scale operation within the Rescale process is grounded in shift operations and fixed-point multiplication, which are more efficient in terms of computation. We first decoupled keys and values using linear interpolation, then approximated the natural exponential function term with Lagrange interpolation, and finally replaced division with shift operations. As a result of these optimizations, during inference, the NSLLM can rely on integer multiplication within a single time step and binary sparse addition across multiple time steps for the entire model. This not only reduces the computational burden, but also makes the model more suitable for deployment in resource-constrained environments, while maintaining high-quality performance on various tasks.

### NSLLM with a layer-wise quantization strategy and sensitivity analysis

As mentioned earlier, we applied quantization techniques in integer spiking neurons, converting floating-point membrane potentials to integer spike values. To address the errors introduced during quantization, especially those from low-bit quantization and the simultaneous quantization of both weights and activation values, we proposed a layer-wise quantization-sensitivity-based approach.

**Figure 2.**
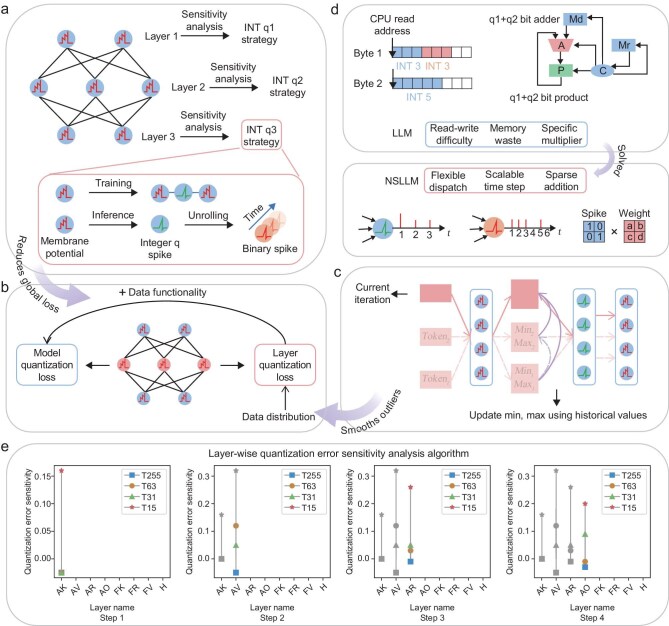
Spike quantization strategy based on layer-wise quantization error sensitivity analysis. (a) Integer quantization strategies (e.g., INT q1–q3) are assigned to each layer based on global sensitivity; training optimizes spiking quantization error, while inference expands binary spikes across virtual time steps. (b) Layer-level error, dominated by data distribution, is reduced by moving-average min–max smoothing, while model-level error, related to data functionality, is minimized via layer-wise sensitivity-based mode selection. (c) Moving-average smoothing mitigates membrane-potential outliers. (d) During inference, traditional LLMs require dedicated multipliers and data alignment for mixed precision, whereas NSLLMs avoid these constraints using event-driven binary spikes across virtual time steps. (e) Layer-wise sensitivity results for the 169M NSLLM select optimal modes (e.g., A1T15, A1T31, A1T63, A1T255); full results, including the 1.5B model, are in Figs S1 and S2.

We first decided on the quantization format and whether outliers should be clipped. To balance computational efficiency and model accuracy, we employed asymmetric quantization for activation values and symmetric quantization for weights. Since the distribution of activation values is uneven with a large dynamic range, it is more complex to quantize them; thus, asymmetric quantization is used to reduce errors [31]. In contrast, the weight distribution is more symmetric and stable, making symmetric quantization feasible, simplifying computation and improving hardware efficiency [32,33]. Furthermore, we considered the impact of outlier clipping on the quantization process. In large-scale model quantization, retaining outliers often increases quantization error, degrading model performance. Traditional outlier clipping methods reduce their impact by removing extreme values [34,35]; however, this may result in the loss of key feature information, negatively affecting overall model performance. To address this, we introduced the moving average MINMAX quantization technique [36] (details can be found in the subsection entitled ‘Spike neuron tools’ in the Methods section below), which dynamically adjusts the quantization range by calculating the moving average of spiking neuron values, as illustrated in Fig. 2c. This approach not only smooths the data distribution and reduces the impact of extreme values, but also avoids information loss, providing a more stable and efficient quantization solution.

We aimed to further reduce the bit-width of quantization without compromising network performance. We believe that the sensitivity of activations to quantization is closely related to the type of layer and its position within the network. In this paper, AK, AV, AR and AO refer to the Key, Value, Receptance and Output layers within the attention block (ATT), while FK, FV and FR represent the Key, Value and Receptance layers within the feed-forward network (FFN). H denotes the Head layer. As shown in Fig. 2b, layer-level quantization loss is affected only by data distribution, whereas model-level quantization loss is influenced by both data functionality and data distribution. Different layers respond differently to model-level quantization; even if some layers have a data distribution more favorable for layer-wise quantization, their critical role in network computation makes them more sensitive to model-level quantization. To evaluate this sensitivity, we used the cross-entropy loss differences between logits and targets under various quantization modes and network layers as sensitivity indicators (details can be found in the subsection entitled ‘Spike neuron tools’ in the Methods section below). Based on this, we proposed a layer-wise sensitivity-based algorithm to determine the optimal quantization mode for each layer in the neural network. As shown in Fig. 2e, we first analyzed the AK layer (the first layer in the block) by applying different quantization modes (e.g. A1T15, A1T31, A1T64, A1T255), which were respectively converted from single-time-step integer spike modes A4T1, A5T1, A6T1 and A8T1 during training, where A denotes the activation bit-width and T represents the number of time steps. We then selected the optimal quantization mode based on the sensitivity analysis (by comparing the error differences between adjacent modes, we set a threshold; if the difference exceeds the threshold, the higher bit-width mode is selected). In the second step, based on the selected quantization mode for the AK layer, we further analyzed the sensitivity of the AV layer, which directly follows the AK layer, and selected its optimal quantization mode. This process was repeated sequentially for each layer, optimizing the quantization mode layer by layer across the network.

From the results in Fig. 2e, we observed significant differences in quantization sensitivity across layers for both the 169M and 1.5B models. In the 169M model, the AK and AR layers exhibited small fluctuations in error across different quantization modes, indicating a certain level of robustness to quantization. However, in the 1.5B model, the quantization error for the AK and AR layers increased in the lower bit-width modes (such as A1T15 or A4T1). This change could be associated with differences in model scale, layer architecture or feature distribution, but the exact mechanism requires further investigation. At the same time, certain layers, such as FV and FR, showed minimal increases in quantization error across different bit-width modes, suggesting that these layers may be better suited to handle different quantization settings under certain conditions. We conducted a series of ablation experiments to compare the mixed–time-step binary spike model (configured as A1T24 for the 169M-parameter model and A1T23 for the 1.5B-parameter model; detailed layer-wise settings are provided in Tables S1 and S2) against four binary-spike configurations: A1T15, A1T24, A1T31 and A1T63. Figure 4a below presents the performance of these models across multiple benchmark tasks. The results show that the mixed–time-step quantization model achieves performance comparable to the A1T63 configuration at both the 169M- and 1.5B-parameter scales. For the 169M model, the mixed–time-step A1T24 binary spike configuration delivers a clear improvement over A1T15, outperforms A1T31 and approaches the performance of A1T63. For the 1.5B model, the mixed–time-step A1T23 configuration yields even more pronounced gains over A1T15 and A1T31, demonstrating superior stability and consistency while matching the performance of A1T63. Therefore, the mixed–time-step binary spike model can maintain a low time-step (or equivalently lower precision) while achieving more substantial performance advantages, particularly in larger-scale models.

It is worth noting that current hardware support for mixed-precision computation has some limitations, especially when dealing with multiple bit-widths, which can lead to compatibility issues. As shown in Fig. 2d (top), traditional LLMs face challenges in this context: low-precision data read-write alignment difficulties, memory waste and the complexity of designing specialized multipliers to accommodate dynamically changing bit-widths make the hardware implementation of mixed-precision computation more complex. In contrast, as shown in Fig. 2d, NSLLMs sidestep the hardware deployment challenges associated with mixed-precision computation by utilizing event-driven binary spike trains to transmit information across virtual time steps. This approach allows NSLLMs to bypass the complexities that arise from mixed-precision computations in traditional LLMs, leading to a more straightforward and efficient deployment on hardware platforms.

### NSLLM with a quantization-assisted sparse strategy exploiting spiking mechanisms

In spiking neural networks, high spike-firing rates significantly increase computational complexity, memory access overhead and energy consumption while weakening the inherent advantage of sparse computation. Each spike triggers membrane potential updates and synaptic weighted accumulation operations. Higher firing rates lead to an increase in computational burdens on neurons, reducing overall efficiency. Moreover, higher spike rates result in a larger volume of spike events and synaptic weight accesses, increasing memory bandwidth requirements and inducing additional data movement overhead. In hardware implementations (such as field-programmable gate arrays or application-specific integrated circuits), frequent memory read and write operations and cross-layer spike propagation further elevate energy consumption, diminishing the energy efficiency advantage of spiking neural networks. Therefore, suppressing the spike-firing rates is crucial in optimizing the computational efficiency and energy management of spiking neural networks.

We further reduced the spike-firing rate in the network by leveraging sparsity. To avoid additional retraining costs, we embedded activation sparsity into the quantization-training process. It should be noted that sparsity and quantization are not independent functions in our design; instead, quantization is employed to significantly enhance the sparsity of spiking neural networks. In large-scale artificial neural networks, activation sparsification techniques have achieved significant progress. However, in NSLLM-based inference, activations are not single integers but mapped to binary spike sequences, shifting focus from single-value sparsity to sequence-level sparsity. Meanwhile, we explored the interplay between sparsification and spiking quantization to minimize the training errors introduced by both, while maximizing their advantages in computational performance and energy-efficiency optimization.

**Figure 3.**
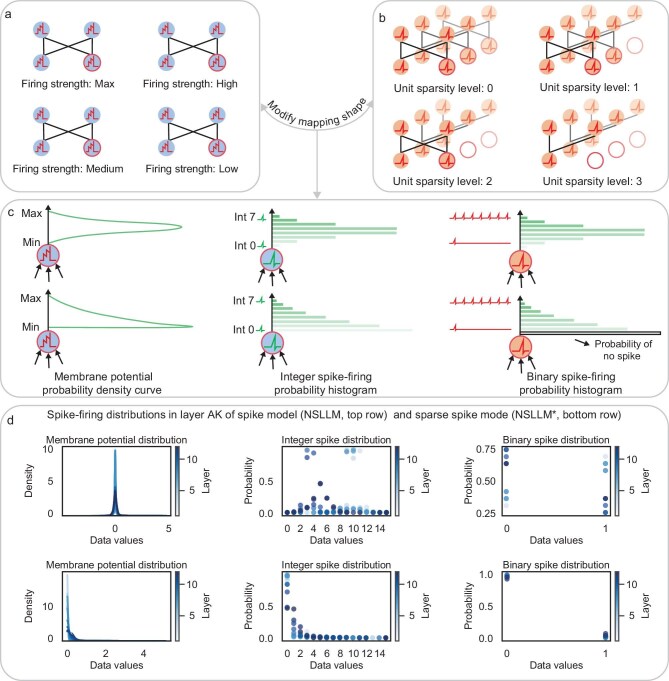
(a) Membrane-potential neurons at different sparsity levels. (b) Binary spiking neurons during inference corresponding to (a), with open circles indicating non-firing neurons. (c) The top row shows the unprocessed bell-shaped membrane-potential distribution, while the bottom row shows the original bell-shaped distribution modified to a half-bell shape. From left to right, each row presents the membrane-potential probability density, the firing probability distribution of different quantized integer spike values, and the firing probability distribution of binary spike trains with different spike counts unfolded over virtual time steps. After the half-bell modification, the dominant range of integer spike firing shifts from middle values to lower values, thereby increasing the likelihood of binary spike trains with sparser spiking activity. (d) Spike-firing distributions in layer AK for the spike model (NSLLM, top) and the sparse spike model (NSLLM*, bottom); additional results are shown in Figs S5–S7.

We also explored the effects of quantization on the sparsity of spiking neural networks alone. During the quantization process, membrane potentials are mapped to integers, which partition the data distribution according to their numerical range. As shown in panels (a) and (b) of Fig. 3, smaller integers contain more zeros when expanded into virtual time steps, thereby exhibiting higher sparsity in the binary spike train. Therefore, we considered whether increasing the proportion of low values in the overall data distribution could enhance the sparsity of the entire dataset. To address this issue, we designed the quantile-shifted rectified linear unit activation function before fake quantization to refine the data distribution through truncation, followed by quantization mapping to integers (details can be found in the subsection entitled ‘Spike neuron tools’ in the Methods section below). We initialized the membrane potential shape modification using the median quantile. In subsequent training, different quantile values can be applied to regulate sparsity. As illustrated in Fig. 3c, this adjustment modifies the probability density distribution of membrane potentials, transforming it from the original bell-shaped distribution into a half-bell-shaped distribution skewed toward lower values. The underlying principle of this transformation is to increase the proportion of low values within the overall data distribution, thereby ensuring that a greater number of values are mapped to smaller integers during the quantization process. Figure 3c presents a sequential representation of the membrane potential distribution, the probability distribution of integer spike firing and the probability distribution of binary spike firing. It can be observed that the density of low-value regions is significantly increased, resulting in a higher proportion of zero values in the binary spike train, thereby enhancing the sparsity of the spiking neural network (for details of spike encoding, see Note S9).

**Figure 4.**
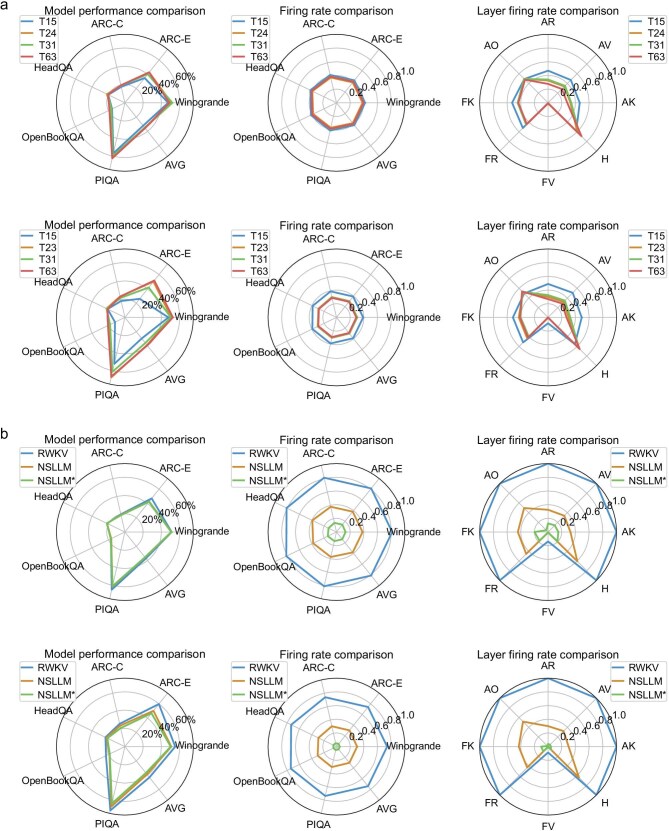
Comparison of zero-shot performance and firing rates for models using different spike quantization and sparsity strategies across multiple benchmark tasks. (a) The performance and spike-firing rates of the 169M (top) and 1.5B (bottom) models under mixed-time-step quantization (A1T24 for 169M and A1T23 for 1.5B) are compared to baseline models with A1T15, A1T31 and A1T63 binary spikes (respectively converted from A4T1, A5T1 and A6T1 during training), all using 4-bit integer weight configurations. The charts display overall performance, spike-firing rates and layer-wise spike-firing rates. (b) The performance and firing rates of the 169M (top) and 1.5B (bottom) models are evaluated using baseline models (RWKV-4), spike models (NSLLM) and sparse spike models (NSLLM*). Both NSLLM and NSLLM* employ the 4-bit integer weight and mixed-time-step binary spike configurations.

In Fig. 4b, we compared the performance and spike-firing rates of the baseline model (RWKV-4), spiking model (NSLLM) and sparse spiking model (NSLLM*) across multiple benchmark tasks (more detailed results and configurations can be found in Tables S1, S2 and S4). Both NSLLM and NSLLM* are based on the quantization configuration of 4-bit integer weights and mixed-time-step binary spikes (A1T24 for 169M and A1T23 for 1.5B). For the 169-million-parameter model, the sparsity of the baseline model is 0.8133. The spiking model performs similarly to the baseline, with a spike-firing rate of 0.3807. The sparse spiking model shows comparable performance to the spiking model, while reducing the spike-firing rate to 0.1355. For the 1.5-billion-parameter model, the sparsity of the baseline model is 0.7392. The spiking model performs similarly to the baseline with a spike-firing rate of 0.3027, while the sparse spiking model shows performance close to the spiking model with the spike-firing rate reduced to 0.0504.

### Multi-dimensional evaluation of the NSLLM following its neuromorphic design

As previously mentioned, the core distinction between the NSLLM and traditional LLMs lies in its objective to bridge the gap between language modeling and neuroscience. This goal also differentiates it from existing studies on spiking language models. In recent years, several works have explored the integration of spiking neural networks (SNNs) into natural language processing tasks, demonstrating the initial feasibility of spiking-based language modeling. For example, models such as SpikingBERT[37], SpikeGPT[38] and SpikeLM[39] focus on small-scale pretrained language models, incorporating spiking computation mechanisms into mainstream architectures and validating the applicability of SNNs in supervised fine-tuning tasks [40,41]. Subsequently, SpikeLLM [42] targets large-scale language modeling tasks by introducing spiking time-step mechanisms into integer-only inference processes to enhance representational capacity, marking a meaningful exploration of the application of spiking computation in the context of LLMs.

These works offer important insights for building larger-scale and more efficient sparse spiking language models, and they also inspire us to pursue a more ambitious goal. However, challenges remain, not only in the higher requirements for time steps and spike sparsity when transitioning from small-scale supervised fine-tuning tasks in vision [43] and language to complex LLM tasks, but also in the significantly different difficulties posed by spiking training and quantization representation. To address this, we are dedicated to realizing truly sparsity-driven and MatMul-free LLMs. Rather than being merely an engineering implementation, our proposed NSLLM represents a scientific exploration toward neuromorphic intelligence. Its core philosophy originates from an interdisciplinary perspective aimed at unifying language modeling and neural mechanisms, using neuromorphic computation as a bridge to foster deep integration between LLMs and neuroscience. Specifically, the NSLLM draws inspiration from biological neural systems, abandoning traditional additive operations and instead adopting spike-driven sparse-weighted operations, and has been further customized with sparsity-driven strategy design and MatMul-free FPGA hardware core design for the LLM, thereby enabling more efficient language inference. In terms of interpretability, the NSLLM is designed from the perspective of computational neuroscience as a neural-population-dynamics modeling framework, capturing coordination patterns among different functional substructures involved in language processing, thereby enhancing the system’s structural transparency and behavioral analyzability.

Guided by the above objectives, we conducted a comprehensive evaluation of the NSLLM across three dimensions: performance, energy efficiency and interpretability. This subsection highlights the comparison between the NSLLM and mainstream LLMs in computational cost and task performance, with subsequent subsections covering its energy efficiency and interpretability evaluations. We compared the performance and multiplication floating-point operations (FLOPs) of NSLLM and NSLLM* with different traditional LLMs (including OPT [23], GPT-Neo [44], Pythia [45], RWKV-4 and TinyLlama [46]) across multiple benchmark tasks, as shown in Table 1. We made comparisons based on two-parameter groupings: one for models with around 100 million parameters and another for models with around 1 billion parameters. It can be observed that the NSLLM reduces FLOPs by 1.8–2.3$\times$ compared to LLMs. For example, the NSLLM with 169 million parameters has 76.38 billion FLOPs, and the NSLLM with 1.5 billion parameters has 1457.91 billion FLOPs. Moreover, the sparse version, NSLLM*, can reduce FLOPs by 5–14$\times$. For example, NSLLM* with 169 million parameters has 27.19 billion FLOPs, and NSLLM* with 1.5 billion parameters has 628.97 billion FLOPs. Compared to LLAMA-2 [2], which achieves strong accuracy but at the cost of significantly higher computational complexity (e.g. 6906.91 billion FLOPs and 13233.87 billion FLOPs for 7B and 13B models, respectively), NSLLM* offers a drastically more efficient solution for resource-constrained settings, reducing FLOPs by up to 9.3$\times$, while still achieving competitive benchmark scores. Even relative to other spiking models like SpikeLLM, our approach is both more efficient and more accurate. For instance, our NSLLM* (1.5B) achieves an average score of 70.54%, outperforming SpikeLLM’s 66.43%, while also requiring fewer FLOPs. In summary, the NSLLM strikes a favorable balance between task performance and FLOPs, illustrating the viability of spike-based computation for large-scale language modeling. More importantly, the NSLLM is not a simplified variant of existing language models, but a neuroscience-inspired rethinking that integrates large-scale language modeling with brain-like computational principles. As a unified architectural framework, the NSLLM is inherently designed to support both energy-efficient computation and interpretable modeling at the neural population level.

**Table 1.** Accuracy of various LLMs across multiple zero-shot benchmark tasks.

		Spike	Time	Params	FLOPs$\downarrow$									
Architecture	Bits	driven	step	(B)	(G)	Winogrande	ARC-E	ARC-C	HeadQA	OpenBookQA	PIQA	BoolQ	HellaSwag	Avg$\uparrow$
OPT	32-32	×	N/A	0.125	145.84	50.28	39.98	22.78	24.98	28.00	62.02	N/A	N/A	38.01
GPT-Neo	32-32	×	N/A	0.125	145.82	50.43	39.39	23.12	25.16	26.20	62.46	N/A	N/A	37.79
Pythia	32-32	×	N/A	0.16	145.86	51.30	39.65	23.63	24.73	26.80	61.92	N/A	N/A	38.01
RWKV-4	32-32	×	N/A	0.169	133.76	51.22	42.42	23.89	25.42	29.80	65.07	N/A	N/A	39.64
**NSLLM**	**4-1**	**✓**	**24**	**0.169**	**76.38**	**50.99**	**39.90**	**23.29**	**26.26**	**29.80**	**59.25**	**N/A**	**N/A**	**38.25**
$\mathrm{NSLLM}^{*}$	**4-1**	**✓**	**24**	**0.169**	**27.19**	**50.83**	**38.30**	**22.78**	**24.76**	**28.80**	**60.45**	**N/A**	**N/A**	**37.65**
TinyLlama	32-32	×	N/A	1.1	1162.33	60.22	46.72	32.51	29.18	37.80	73.29	N/A	N/A	46.62
OPT	32-32	×	N/A	1.3	1445.46	59.51	50.97	29.52	26.00	33.40	72.47	N/A	N/A	45.31
GPT-Neo	32-32	×	N/A	1.3	1445.43	54.93	50.21	25.85	27.86	33.60	71.06	N/A	N/A	43.92
Pythia	32-32	×	N/A	1.4	1445.52	57.54	53.87	28.58	27.35	33.20	71.00	N/A	N/A	45.26
RWKV-4	32-32	×	N/A	1.5	1445.47	55.17	53.32	29.86	27.61	34.40	71.44	N/A	N/A	45.30
**NSLLM**	**4-1**	**✓**	**23**	**1.5**	**628.97**	**50.83**	**45.29**	**26.79**	**26.22**	**31.00**	**66.59**	**N/A**	**N/A**	**41.12**
$\mathrm{NSLLM}^{*}$	**4-1**	**✓**	**23**	**1.5**	**104.72**	**50.20**	**42.13**	**23.55**	**25.89**	**28.80**	**63.66**	**N/A**	**N/A**	**39.04**
LLAMA-2	16-16	×	N/A	7	6906.31	69.22	74.54	46.33	N/A	N/A	78.84	77.74	75.97	70.44
SpikeLLM	4-4	×	1.2	7	1035.95	59.43	62.29	36.01	N/A	N/A	72.47	69.48	64.74	60.74
$\mathrm{NSLLM}^{*}$	**4-1.5**	✓	**8**	**7**	**745.88**	**66.14**	**69.65**	**41.21**	**N/A**	**N/A**	**75.84**	**74.01**	**71.75**	**66.43**
LLAMA-2	16-16	×	N/A	13	13 233.87	71.74	77.48	49.23	N/A	N/A	80.63	80.73	79.37	80.69
SpikeLLM	4-4	×	1.2	13	1985.08	65.51	69.53	41.21	N/A	N/A	75.79	74.31	71.51	66.31
$\mathrm{NSLLM}^{*}$	**4-1.5**	✓	**8**	**13**	**1429.26**	**69.85**	**74.12**	**46.16**	**N/A**	**N/A**	**78.51**	**78.26**	**76.36**	**70.54**

In the Bits column, the two numbers denote the bit-width of the weight and activity, respectively, where 1 indicates binary values (0/1) and 1.5 indicates ternary values (–1/0/1). ‘$^{*}$’ indicates the use of a spiking sparsity strategy. We use an optimally customized mixed time-step configuration for different models (see Tables S1 and S2 for details). FLOPs are calculated based on the result of all time steps (for ANN, we treat time steps = 1). For additional results, we refer the reader to Note S10.

### NSLLM with a billion-scale MatMul-free hardware design

**Figure 5.**
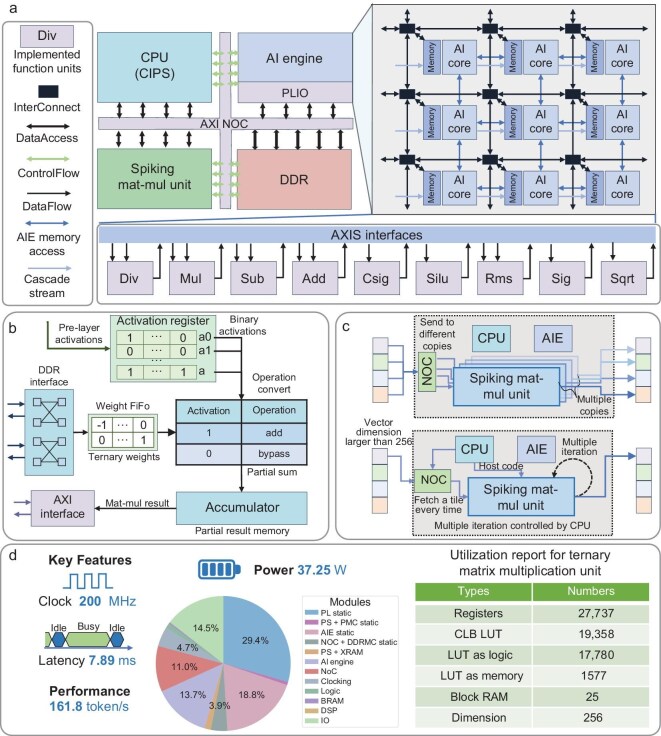
RTL implementation for running NSLLM token generation on an FPGA accelerator. (a) Overall architecture of the FPGA implementation. The AXI NOC transfers data to computing units, with CIPS handling system control. The AI engine (AIE) performs vector computations, while the MatMul-free unit executes ternary operations. The design supports efficient out-of-order execution with specialized algorithms for enhanced precision and reduced rounding errors. (b) Efficient ternary non-matrix multiplication: activations stored in Vector Register. The ternary matrix unit reads ternary weights and converts MACs to ACs for multiplication. (c) Execution of a MatMul-free unit for larger *d*. Top: utilization of multiple units for parallel computation. Bottom: the unit achieves the required computation in multiple iterations under control of the CPU. (d) FPGA implementation results and Utilization report. We report key results of the FPGA implementation and resource Utilization of the MatMul-free unit ($d=256$). Deploying our 1.5B NSLLM on the FPGA results in a total power consumption of only 27.35 W, with dynamic power as low as 13.849 W.

Through a series of optimization designs within the NSLLM architecture, we not only significantly reduced memory requirements, but also transformed multiplication operations into addition and subtraction, further enhancing computational efficiency, which aligns with the energy efficiency advantages of spiking neural networks. To validate the energy efficiency of the NSLLM, we developed a customized hardware design tailored to its event-driven nature characterized by sparse binary spikes during inference, integrated with innovative algorithmic optimizations, and extended our previous work initially published on arXiv [47] (now fully incorporated into this paper), as shown in Fig. 5. In terms of hardware implementation, we designed a dedicated ternary hardware core to perform non-matrix multiplication operations, based on binary $\lbrace 0, 1\rbrace$ or ternary $\lbrace -1, 0, 1\rbrace$ sets for activations and weights (details can be found in Note S11). This unit maximizes computational efficiency by converting multiplication operations into addition, subtraction or bypass operations depending on the weight (e.g. an activation of 1 and a weight of −1 triggers a subtraction). To validate the effectiveness of this approach and assess its hardware compatibility, we implemented the solution on AMD’s Versal (VCK190) FPGA platform.

We utilized the AI engine (Xilinx’s AIE) to execute the vector-vector operations such as row-wise addition, row-wise subtraction, row-wise multiplication, row-wise division, root mean square, etc, while utilizing the functional acceleration flow to execute the MatMul-free operation. The AIE compute kernels are implemented in C++ and are then represented as nodes in the adaptive data-flow graphs, connected to the DDR via programmable logic inputs/outputs (PLIOs). PLIOs are working as IO channels (data movers) to feed and fetch data to and from the AIE (moves data between DDR memory and the AI engine). Each channel runs at 5 GB/s and has a 128-bit$\times$4096 URAM buffer that stores segments of input data or results before receiving or sending them to the DDR. In addition to this, each channel has counters to count the latency between the cycle at which it sends or fetches data to or from AIE and the cycle at which it finishes all sending/fetching operations. AMD’s AIE is a computational core that has multiple vector processing units connected in a consistent two-dimensional array (similar to systolic arrays), making it highly efficient for vectorized computation at a low power budget, hence the choice of Versal device. VCK190’s AIE showed high performance running our row-wise operations (addition, subtraction, multiplication, division, square root and root mean square).

While the MatMul-free unit could also be designed on the AI engine, utilizing its power of parallel processing, we chose to design it as a hardware kernel controlled directly by the VCK190’s CPU. The reasons for this include acceleration and computation optimization, as it requires higher parallelism than that offered in AIE, efficiency in utilizing sparsity and quantized model weights, and the need for frequent use of this kernel. This single MatMul-free unit runs at 200 MHz clock frequency and completes, in parallel, a forward pass of a block in $5.885\, \mu$s for an embedding dimension $d=256$. For larger *d*, we can either utilize the kernel in multiple iterations to achieve the required computation, with the host code running on the CPU to control the unit, or we can even utilize multiple copies of this unit in the hardware, enabling parallel computation. The choice of $d=256$ in this unit is to balance the memory bandwidth as well as the resource utilization of the FPGA implementation.

Moreover, when deploying the proposed NSLLM on the VCK190 FPGA (the specific model corresponds to the NSLLM* in Table S10), we achieved a significant reduction in total power consumption to 27.35 W, with dynamic power as low as 13.849 W. Additionally, MatMul operations were completely eliminated, while maintaining robust performance at the billion-parameter scale. For a 24-layer model with a sequence length of 1024 and an embedding dimension of 2048, we obtained an inference memory usage of 946 MiB and an inference throughput of 161.8 tokens/s. In comparison, RWKV-4 on the A800 GPU (with the same configuration) exhibits a total power consumption of 328.60 W, including dynamic power of 274.93 W, an inference memory usage of 20 182 MiB and an inference throughput of 74.7 tokens/s (batch size = 1, corresponding to the typical latency-critical edge scenario). Our deployment demonstrates improvements in dynamic power efficiency, memory usage and inference throughput by 19.8$\times$, 21.3$\times$ and 2.2$\times$, respectively. These results offer valuable insights for the design of next-generation neuromorphic chips for large-scale models.

### NSLLM as a neural population for better interpretability

**Figure 6.**
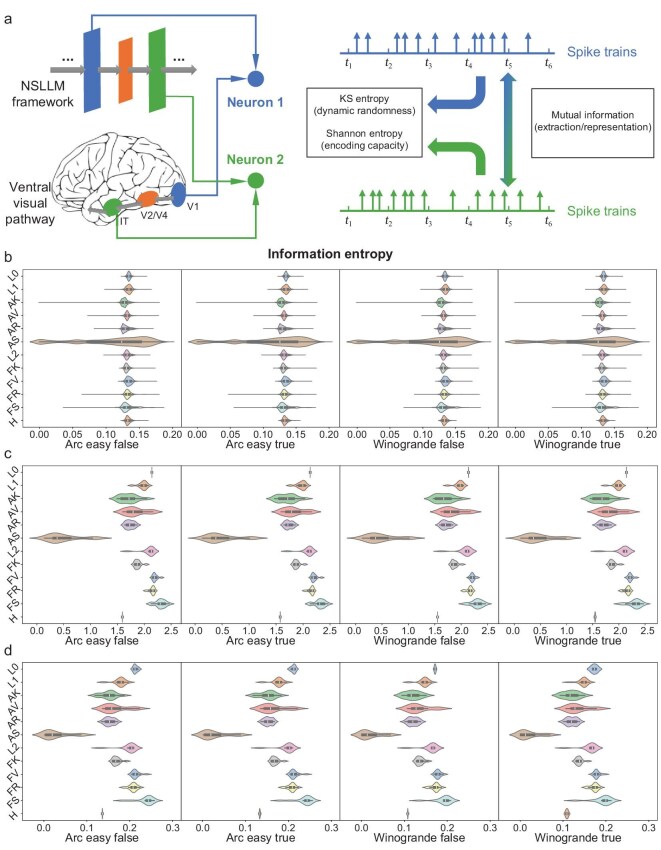
The neural dynamical analysis of NSLLM outcomes. (a) The analysis framework for the NSLLM as a neural population. Based on the neuron spike trains acquired from layers (in the NSLLM) or regions (in neural systems), we can assess the dynamic randomness and encoding capacity within layers or regions, and representation capability and information difference between different layers or regions. (b) The mean KS entropy of different layers. (c) The mean Shannon entropy of different layers. (d) The MI between the initial layer ‘quant’ and other layers. The corresponding results of the significance test (*t*-test, two-tailed) can be found in Fig. S10.

As previously mentioned, our framework enables us to analyze and interpret information processing in LLMs with theoretical neuroscience methods. To demonstrate, we combined the framework summarized in Fig. 1 with an approach developed in our previous work [24]. We demonstrated how to model LLMs as large-scale populations of spiking neurons, where each neuron receives information directly from its pre-synaptic neurons or the external environment, thereby exhibiting non-linear and complex neural activities. On the one hand, this modeling method enables the analysis of neuronal dynamic properties, such as dynamic randomness, as quantified by the Kolmogorov–Sinai entropy (KS entropy) [24,48]. On the other hand, it allows the characterization of each neuron as an information channel, thereby facilitating the quantification of its information processing capacity, for instance, the amount of properly encoded information input as measured by mutual information (MI) [25,49]. These details can be found in the subsection entitled ‘Neural dynamics and information analysis’ in the Methods section below. By combining these two aspects, we can explore how the dynamic properties of individual neurons in the NSLLM determine their information processing capacity, and to some extent, such analysis allows us to study the NSLLM as a neural population, as shown in Fig. 6a.

**Figure 7.**
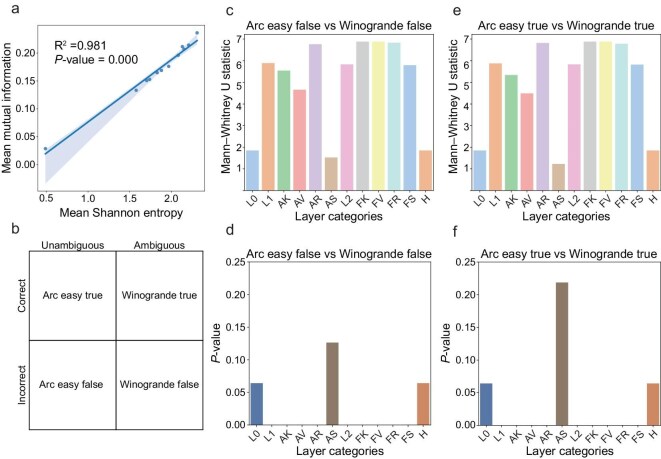
The statistical analysis of NSLLM dynamics. (a) The Pearson correlation analysis results for corresponding metrics on the dataset ‘arc easy true’. More results on other datasets can be found in Fig. S12. (b) The classification of four datasets based on two sets of factors: sentences with unambiguous or ambiguous information, and correct or incorrect corresponding judgments. (c and d) The Mann–Whitney U test results of normalized MI of each layer between datasets ‘arc easy false’ and ‘winogrande false’. (e and f) The Mann-Whitney U test results of normalized MI of each layer between datasets ‘arc easy true’ and ‘winogrande true’.

Specifically, we designed a representative experiment in which the NSLLM processes the sentences with or without ambiguous information and makes judgments about these sentences. Based on the correctness of judgments, we can further distinguish among four conditions, as shown in Fig. 7a. The associated information processing in the NSLLM can be regarded as effective when the judgments are correct; otherwise, the information processing is regarded as ineffective. By applying the analysis methods introduced above, we can explore how different language materials are processed from both dynamical and informational perspectives. This approach further provides potential pathways for interpreting model prediction behaviors through a neuroscience-inspired framework.

As shown in Fig. 6b, we presented the mean KS entropy of different layers, showing that neurons from all layers exhibit similar dynamic randomness except for the ‘att.sigmoid’ (AS) layer. The distinct dynamic behaviors in the AS layer indicate that the neurons in this layer play a unique role in sparse representation in information processing. We then evaluated the encoding capacity of neurons from different layers by comparing their mean Shannon entropy. As shown in Fig. 6c, the ‘ffn.sigmoid’ (FS) layer outperforms the other layers, suggesting that neurons in this layer can convey more information, while the AS layer exhibits the lowest average Shannon entropy, indicating a lower information capacity. This may indicate that the sparse spiking activity in the AS layer limits the amount of information that can be encoded. In Fig. 6d, we presented the mean normalized MI between the initial layer ‘quant’ and other layers, representing each layer’s capability to extract and represent information relative to the initial layer. Notably, as shown in Fig. 7a, the MI results positively correlate with the Shannon entropy results in Fig. 6d. This result suggests that layers with higher encoding capacity, as presented by Shannon entropy, are also more effective in preserving information originating from the model’s initial layer (input layer).

To assess whether the model can differentiate the datasets containing sentences with or without ambiguous information, we performed Mann–Whitney U tests on the normalized MI of each layer across the corresponding datasets. As shown in Fig. 7b, the four datasets are classified by two sets of factors: sentences with unambiguous or ambiguous information, and correct or incorrect corresponding judgments. The Mann–Whitney U test results, as illustrated in Fig. 7(c–f), suggest that the model can effectively distinguish between datasets with or without ambiguous information, exhibiting statistically significantly higher normalized MI in most middle layers for the datasets without ambiguous information, regardless of whether correct judgments are provided. The results indicate that while processing language materials with unambiguous texts, the model presents more effective information processing and representation capability compared to those with ambiguous information. However, the model cannot effectively distinguish between datasets that make correct judgments or not, based on the normalized MI results. This suggests that the information representation of the model is not substantially different while processing the correctness of judgments, indicating less sensitivity than when processing the ambiguity of sentences. More details can be found in Fig. S11.

In conclusion, the AS layer, with its sparse spiking activity, exhibits lower information capacity and higher information differences compared to other layers, while the FS layer exhibits superior performance in information extraction and representation. The observed positive correlation between Shannon entropy and normalized MI further supports this finding, suggesting that layers with higher information capacity experience reduced information distortion during network propagation. Moreover, the model shows pronounced sensitivity to language materials with or without ambiguous information, displaying significantly higher representational capacity when processing unambiguous sentences. Additional analysis results can be found in Figs S10–S12. Notably, in the analysis above, the spike-train representation of data enabled detailed dynamical analysis with substantially reduced experimental trials and data volume. Meanwhile, the NSLLM framework provides intuitive and biologically interpretable insights, advancing the understanding of both artificial systems, such as large language models, and biological systems, such as complex nervous networks, from a unified new perspective.

## DISCUSSION

In this work, we established a unified framework by demonstrating a neuromorphic alternative to traditional LLMs. The proposed framework not only advances the state of the art in energy-efficient AI, but also provides a novel perspective on AGI development, where biological efficiency, interpretability and computational power converge to create more adaptable and sustainable intelligent systems. From a neuroscientific perspective, the adoption of SNNs in large-scale AI models brings us closer to understanding the fundamental principles of brain-like computation. Unlike conventional deep learning models that continuously process information, the discrete, event-driven nature of spiking neurons mirrors biological signal processing, where neural activity is driven by meaningful events rather than redundant background computation. This could lead to new insights into cognitive efficiency, potentially enabling AI systems to exhibit more human-like adaptability and resource allocation. Furthermore, the introduction of attention mechanisms in neuromorphic models suggests that high-level cognitive functions, such as selective information processing and hierarchical decision-making, could be efficiently implemented using neuromorphic architectures, offering a biologically plausible path toward AGI.

Interpretability is one of the critical aspects of AGI development. Current LLMs function as black boxes, making it difficult to decipher their decision-making processes. Neuromorphic computing, by contrast, offers a more transparent computational architecture through neural populations. The event-driven nature of SNNs allows the tracing of information flow at the neuronal level, making it easier to analyze, debug and even intervene in decision processes. By integrating these properties into LLMs, the NSLLM could serve as a stepping stone toward self-explainable AI models that provide explicit justifications for their outputs, a crucial feature for applications in critical fields such as healthcare, finance and autonomous systems. Moreover, energy efficiency is a fundamental constraint in AI scalability, especially in edge computing and real-time interactive systems. While LLMs have demonstrated unprecedented performance in the field of natural language processing, their exponential growth in model size and computational complexity poses severe energy challenges. Neuromorphic approaches inherently reduce redundancy through sparse and event-driven processing, potentially making AGI systems viable on low-power hardware, embedded systems and self-powered devices. In the long run, AGI with neuromorphic chips could lead to AI systems that operate sustainably within biological and ecological constraints, much like the human brain.

Looking ahead, the convergence of neuroscience, AI and energy-efficient hardware engineering is crucial for AGI development. Neuromorphic LLMs represent just the beginning of this integration. Future advancements might explore hybrid systems that combine the strengths of both deep learning and neuromorphic processing. These systems could leverage mixed-precision computation to fine-tune the trade-off between model accuracy and energy consumption, while simultaneously incorporating higher-level cognitive functions like reasoning, memory consolidation and meta-learning. The potential for AGI to emerge from these cross-disciplinary efforts offers not only a technical challenge but a philosophical one: what would it mean for AI to develop cognitive processes closer to those of humans, and what ethical considerations would arise from creating machines that can learn, adapt and explain their actions like human minds do? The answers to these questions could shape the trajectory of AGI, making the path from technical innovation to societal impact as important as the breakthrough itself.

## METHODS

### Spiking neuron models

Spiking neurons are the fundamental computational units of SNNs. They accumulate membrane potential over time and emit binary spikes when a threshold is reached, simulating biological neurons. We chose the LIF spiking neuron model for its biological interpretability and computational efficiency [8,50]. However, the quantization error of binary spikes limits their representational capability. To address this, we propose a flexible integer spiking neuron model to reduce quantization errors. This neuron processes integer values during training and converts them into 0/1 spikes during inference. By quantizing and dequantizing the membrane potential, we learn pulse quantization errors, optimizing performance. Unlike traditional SNN training [51,52], we use gradient descent for back-propagation, optimizing training time while maintaining spike output during inference (for details and formulas, see Note S1).

### Spike linear attention

Traditional Transformers use the soft attention mechanism, but their quadratic complexity limits efficiency when processing long sequences. Linear attention optimizes the computation, reducing the complexity to $O(Ld)$, significantly improving efficiency. We propose a spiking linear attention mechanism based on relative position encoding and key-value separation techniques of the RWKV architecture. We found that RWKV and spiking neural networks share similarities in spatiotemporal information aggregation, allowing RWKV’s linear attention mechanism to efficiently adapt to spiking neural networks, leveraging the strengths of both while maintaining computational efficiency (for details and formulas, see Note S2).

### Spike neuron tools

In the spiking quantization process, we adopted several techniques to improve quantization performance and computational efficiency. First, we used moving averages to smooth the membrane potential and reduce the impact of extreme values. Next, we designed a hierarchical quantization-sensitivity formula to evaluate activation-quantization sensitivity and errors between different layers, optimizing the configuration of mixed time steps. Simultaneously, we integrated an automatic mixed-precision strategy into the quantization-training process. In spiking sparse quantization, we designed a quantile ReLU function to shift the membrane potential quantization probability toward lower pulse counts, increasing the proportion of zero values in the binary spike train and thus significantly reducing the firing rate. Finally, we adopted virtual time-step expansion to convert the spikes into binary sequences and combined the time steps with firing rates to evaluate the operation count, improving computational efficiency (for details and formulas, see Note S3). During hardware deployment, we chose ternary weights and activations, and designed a MatMul-free hardware core that replaces traditional matrix multiplication with simplified addition operations. This approach avoids hardware-unfriendly matrix multiplications and optimizes the solution for hardware deployment.

### Neural dynamics and information analysis

In neural dynamics and information analysis, we transform neuron spike trains into probability distributions to derive entropy metrics that evaluate the temporal dynamics of the neuronal encoders in the NSLLM. The mean Shannon entropy of each layer reflects information richness and diversity, with higher entropy correlating with changes in the brain’s functional states. To assess representational capability, we calculate normalized mutual information, quantifying shared information between neural stimuli (input layer) and responses (hidden layer). We use KS entropy to evaluate the complexity or randomness of neural dynamics across layers, with higher KS values indicating more chaotic and unpredictable behavior, possibly related to cognitive states (for details and formulas, see Note S14).

## Supplementary Material

nwaf551_Supplemental_File

## Data Availability

All data used in this paper are publicly available and can be accessed at https://huggingface.co/datasets/allenai/winogrande for the Winogrande dataset, https://huggingface.co/datasets/allenai/ai2_arc for the ARC Easy and ARC Challenge datasets, https://huggingface.co/datasets/allenai/openbookqa for the OpenBookQA dataset, https://huggingface.co/datasets/ybisk/piqa for the PIQA dataset, https://huggingface.co/datasets/dvilares/head_qa for the HeadQA dataset, https://huggingface.co/datasets/google/boolq for the BoolQ dataset and https://huggingface.co/datasets/Rowan/hellaswag for the HellaSwag dataset. The source code is publicly available at https://github.com/BICLab.

## References

[1] OpenAI . ChatGPT: Optimizing Language Models for Dialogue. https://openai.com/index/chatgpt/?utm_523 source=chatgpt.com (20 November 2025, date last accessed).

[2] Touvron H, Martin L, Stone K et al. Llama 2: open foundation and fine-tuned chat models [preprint]. arXiv: 2307.09288.

[3] Maslej N, Fattorini L, Brynjolfsson E et al. Artificial intelligence index report 2023 [preprint]. arXiv: 2310.03715.

[4] Kwon W, Li Z, Zhuang S et al. Efficient memory management for large language model serving with PagedAttention. In: Proceedings of the 29th Symposium on Operating Systems Principles. New York: Association for Computing Machinery, 2023, 611–26. 10.1145/3600006.3613165

[5] Bricken T, Templeton A, Batson J et al. Towards monosemanticity: Decomposing language models with dictionary learning. https://transformer-circuits.pub/2023/monosemantic-features/index.html (20 November 2025, date last accessed).

[6] Zhao H, Chen H, Yang F et al. Explainability for large language models: A survey. ACM Trans Intell Syst Technol 2024; 15: 20.

[7] Mehonic A, Kenyon AJ. Brain-inspired computing needs a master plan. Nature 2022; 604: 255–60. 10.1038/s41586-021-04362-w 35418630

[8] Roy K, Jaiswal A, Panda P. Towards spike-based machine intelligence with neuromorphic computing. Nature 2019; 575: 607–17. 10.1038/s41586-019-1677-2 31776490

[9] Yu L, Yu Y. Energy-efficient neural information processing in individual neurons and neuronal networks. J Neurosci Res 2017; 95: 2253–66. 10.1002/jnr.24131 28833444

[10] Doshi-Velez F, Kim B. Towards a rigorous science of interpretable machine learning [preprint]. arXiv: 1702.08608.

[11] Brown T, Mann B, Ryder N et al. Language models are few-shot learners. In: Proceedings of the 34th International Conference on Neural Information Processing Systems. Red Hook, NY: Curran Associates 2020, 1877–901.

[12] Lee C, Jin J, Kim T et al. OWQ: lessons learned from activation outliers for weight quantization in large language models [preprint]. arXiv: 2306.02272.

[13] Lin J, Tang J, Tang H et al. AWQ: activation-aware weight quantization for LLM compression and acceleration [preprint]. arXiv: 2306.00978.

[14] Ribeiro MT, Singh S, Guestrin C. “Why should i trust you?”: explaining the predictions of any classifier. In: Proceedings of the 22nd ACM SIGKDD International Conference on Knowledge Discovery and Data Mining. New York: Association for Computing Machinery, 2016, 1135–44.

[15] Lundberg SM, Lee SI. A unified approach to interpreting model predictions. In: Proceedings of the 31st International Conference on Neural Information Processing Systems. Red Hook, NY: Curran Associates, 2017, 4768–77.

[16] Chefer H, Gur S, Wolf L. Transformer interpretability beyond attention visualization. In: IEEE/CVF Conference on Computer Vision and Pattern Recognition. Piscataway, NJ: IEEE Press, 2021, 782–91.

[17] Guidotti R. Counterfactual explanations and how to find them: literature review and benchmarking. Data Min Knowl Discov 2024; 38: 2770–824. 10.1007/s10618-022-00831-6

[18] Montavon G, Binder A, Lapuschkin S et al. Layer-wise relevance propagation: an overview. In: Samek W, Montavon G, Vedaldi A et al. (eds.) Explainable AI: Interpreting, Explaining and Visualizing Deep Learning. Cham: Springer, 2019, 193–209. 10.1007/978-3-030-28954-6_10

[19] Ali A, Schnake T, Eberle O et al. XAI for transformers: better explanations through conservative propagation. In Proceedings of the 39th International Conference on Machine Learning (Proc Mach Learn Res 162), JMLR, 2022, 435–51.

[20] Kathail V. Xilinx vitis unified software platform. In: Proceedings of the 2020 ACM/SIGDA International Symposium on Field-Programmable Gate Arrays. New York: Association for Computing Machinery, 2020, 173–4. 10.1145/3373087.3375887

[21] Touvron H, Lavril T, Izacard G et al. LLaMA: open and efficient foundation language models [preprint]. arXiv: 2302.13971.

[22] Peng B, Alcaide E, Anthony QG et al. RWKV: reinventing RNNs for the transformer era. In: Findings of the Association for Computational Linguistics: EMNLP 2023. Stroudsburg, PA: Association for Computational Linguistics, 2023, 14048–77. 10.18653/v1/2023.findings-emnlp.936

[23] Zhang S, Roller S, Goyal N et al. OPT: open pre-trained transformer language models [preprint]. arXiv: 2205.01068.

[24] Tian Y, Li G, Sun P. Bridging the information and dynamics attributes of neural activities. Phys Rev Res 2021; 3: 043085. 10.1103/PhysRevResearch.3.043085

[25] Cover TM. Elements of Information Theory. New York: John Wiley and Sons, 1999.

[26] Wu Z, Zhang H, Lin Y et al. LIAF-Net: leaky integrate and analog fire network for lightweight and efficient spatiotemporal information processing. IEEE Trans Neural Netw Learn Syst 2021; 33: 6249–62. 10.1109/TNNLS.2021.3073016 33979292

[27] Maass W. Networks of spiking neurons: the third generation of neural network models. Neural Netw 1997; 10: 1659–71. 10.1016/S0893-6080(97)00011-7

[28] Vaswani A, Shazeer N, Parmar N et al. Attention is all you need. In: Proceedings of the 31st International Conference on Neural Information Processing Systems. Red Hook, NY: Curran Associates, 2017, 6000–10.

[29] Gu A, Dao T. Mamba: Linear-time sequence modeling with selective state spaces. First Conference on Language Modeling, Philadelphia, PA, 7–9 October 2024.

[30] Wang S, Li BZ, Khabsa M et al. Linformer: self-attention with linear complexity [preprint]. arXiv: 2006.04768.

[31] Liu Z, Oguz B, Zhao C et al. LLM-QAT: data-free quantization aware training for large language models [preprint]. arXiv: 2305.17888.

[32] Xiao G, Lin J, Seznec M et al. SmoothQuant: accurate and efficient post-training quantization for large language models. In: Proceedings of the 40th International Conference on Machine Learning (Proc Mach Learn Res 202), JMLR, 2023, 38087–99.

[33] Lin J, Tang J, Tang H et al. AWQ: activation-aware weight quantization for on-device LLM compression and acceleration. In: Proceedings of the Seventh Annual Conference on Machine Learning and Systems. MLSYS, 2024, 87–100.

[34] Wei X, Zhang Y, Li Y et al. Outlier suppression+: accurate quantization of large language models by equivalent and effective shifting and scaling. In: Proceedings of the 2023 Conference on Empirical Methods in Natural Language Processing. Stroudsburg, PA: Association for Computational Linguistics, 2023, 1648–65. 10.18653/v1/2023.emnlp-main.102

[35] Shao W, Chen M, Zhang Z et al. OmniQuant: omnidirectionally calibrated quantization for large language models. 12th International Conference on Learning Representations, Vienna, Austria, 7–11 May 2024.

[36] Jacob B, Kligys S, Chen B et al. Quantization and training of neural networks for efficient integer-arithmetic-only inference. In: IEEE/CVF Conference on Computer Vision and Pattern Recognition. Piscataway, NJ: IEEE Press, 2018, 2704–13.

[37] Bal M, Sengupta A. SpikingBERT: distilling BERT to train spiking language models using implicit differentiation. In: Proceedings of the 38th AAAI Conference on Artificial Intelligence. Washington, DC: AAAI Press, 2024, 10998–06. 10.1609/aaai.v38i10.28975

[38] Zhu RJ, Zhao Q, Li G et al. SpikeGPT: generative pre-trained language model with spiking neural networks. Trans Mach Learn Res 2024.

[39] Xing X, Zhang Z, Ni Z et al. SpikeLM: towards general spike-driven language modeling via elastic bi-spiking mechanisms. In: Proceedings of the 41st International Conference on Machine Learning (Proc Mach Learn Res 235), JMLR, 2024, 54698–714.

[40] Socher R, Perelygin A, Wu J et al. Recursive deep models for semantic compositionality over a sentiment treebank. In: Proceedings of the 2013 Conference on Empirical Methods in Natural Language Processing. Stroudsburg, PA: Association for Computational Linguistics, 2013, 1631–42. 10.18653/v1/D13-1170

[41] Wang A, Singh A, Michael J et al. GLUE: a multi-task benchmark and analysis platform for natural language understanding. In: Proceedings of the 2018 EMNLP Workshop BlackboxNLP: Analyzing and Interpreting Neural Networks for NLP. Stroudsburg, PA: Association for Computational Linguistics, 2018, 353–5. 10.18653/v1/W18-5446

[42] Xing X, Gao B, Liu Z et al. SpikeLLM: scaling up spiking neural network to large language models via saliency-based spiking. 13th International Conference on Learning Representations, Singapore, 24–28 April 2025.

[43] Yao M, Qiu X, Hu T et al. Scaling spike-driven transformer with efficient spike firing approximation training. IEEE Trans Pattern Anal Mach Intell 2025; 47: 2973–90. 10.1109/TPAMI.2025.3530246 40031207

[44] Black S, Gao L, Wang P et al. GPT-Neo: Large Scale Autoregressive Language Modeling with Mesh-TensorFlow. 10.5281/zenodo.5297715 (20 November 2025, date last accessed).

[45] Biderman S, Schoelkopf H, Anthony Q et al. Pythia: a suite for analyzing large language models across training and scaling. In: Proceedings of the 40th International Conference on Machine Learning (Proc Mach Learn Res 202), JMLR, 2023, 2397–430.

[46] Zhang P, Zeng G, Wang T et al. TinyLlama: an open-source small language model. arXiv: 2401.02385.

[47] Zhu RJ, Zhang Y, Sifferman E et al. Scalable MatMul-free language modeling. arXiv: 2406.02528.

[48] Engelken R, Wolf F, Abbott LF. Lyapunov spectra of chaotic recurrent neural networks. Phys Rev Res 2023; 5: 043044. 10.1103/PhysRevResearch.5.043044

[49] Dayan P, Abbott LF. Theoretical Neuroscience: Computational and Mathematical Modeling of Neural Systems. Cambridge, MA: MIT Press, 2001.

[50] Pei J, Deng L, Song S et al. Towards artificial general intelligence with hybrid tianjic chip architecture. Nature 2019; 572: 106–11. 10.1038/s41586-019-1424-8 31367028

[51] Li Y, Deng S, Dong X et al. A free lunch from ANN: towards efficient, accurate spiking neural networks calibration. In: Proceedings of the 38th International Conference on Machine Learning (Proc Mach Learn Res 139), JMLR, 2021, 6316–25.

[52] Bu T, Fang W, Ding J et al. Optimal ANN-SNN conversion for high-accuracy and ultra-low-latency spiking neural networks. 10th International Conference on Learning Representations, Virtual, 25–-29 April 2022.

