# Supplementary material for: Neuromorphic spike-based large language model
Source: Natl Sci Rev. 2025 Dec 4;13(4):nwaf551. doi: 10.1093/nsr/nwaf551 (PMC12906346; doi:10.1093/nsr/nwaf551)
Supplement: nwaf551_Supplemental_File [file nwaf551_supplemental_file.pdf]

Supplementary Materials for Neuromorphic Spike-based Large Language Model

Han Xu[1,2,3,†], Xuerui Qiu[1,4,†], Yunhui Xu[5,†], Mohammed E. Elbtity[6,†], Peng Zhou[7,†], Yang Tian[5], Rui-Jie Zhu[8], Jiahong Zhang[1,2], Shaowei Gu[1,4], Yuqi Pan[1], Yuhong Chou[9], Qinghao Wen[1,10], Man Yao[1], Jiangbo Qian[11], Yonghong Tian[3], Lei Ma[3,12], Tiejun Huang[3,12], Jason K. Eshraghian[8], Bo Xu[1,2*], and Guoqi Li[1,2,13*]

[1]Institute of Automation, Chinese Academy of Sciences, Beijing, China
[2]School of Artificial Intelligence, University of Chinese Academy of Sciences, Beijing, China
[3]Beijing Academy of Artificial Intelligence, Beijing, China
[4]School of Future Technology, University of Chinese Academy of Sciences, Beijing, China
[5]Department of Psychology, Tsinghua University, Beijing, China
[6]Advanced Micro Devices, Inc, The United States of America
[7]LuxiTech, Shenzhen, China
[8]University of California, Santa Cruz, The United States of America
[9]The Hong Kong Polytechnic University, Hongkong, China
[10]School of Aerospace, Mechanical and Mechatronic Engineering, The University of Sydney, Australia
[11]Faculty of Electrical Engineering and Computer Science, Ningbo University, Zhejiang, China
[12]School of Computer Science, Peking University Beijing, Beijing, China
[13]Key Laboratory of Brain Cognition and Brain-inspired Intelligence Technology, Beijing, China
[†]These authors contributed equally to this work.
[*]Corresponding authors (guoqi.li@ia.ac.cn; xubo@ia.ac.cn)

Supplementary Note 1: Details of spiking neuron models method

Spiking neurons are the fundamental computation units of SNN, communicating via spikes coded in binary activations, which closely mimic the behaviors of biological neurons. The key difference between traditional artificial neurons and spiking neurons is that the latter takes the dynamics of the temporal dimension into account. The dynamics of a spiking neuron can be simply described as: accumulating membrane potential over time from either the environment (via input information to the network) or from internal communications (typically via spikes from other neurons in the network) When the membrane potential reaches a certain threshold, the neuron fires spikes and updates the membrane potential. Qualitative scientific investigation of the membrane voltage of neurons has been carried out since 1907[53]. In contrast to the many-variable and intricate Hodgkin-Huxley (H-H) model[54], the leaky-integrate-and-fire (LIF) neuron model has a significantly reduced computational demand and is commonly recognized as the simplest model among all popular neuron models while retaining biological interpretability[8,50]. In this paper, we adopted the LIF spiking neuron model and translated it to an iterative expression with the Euler method[55]. Mathematically, the LIF-SNN layer can be described as an explicitly iterable version for better computational traceability:

$$\mathbf{u}^{(\ell)}[t] = \mathbf{h}^{(\ell)}[t-1] + f(\mathbf{W}^{(\ell)}, \mathbf{x}^{(\ell-1)}[t]) \tag{1}$$

$$\mathbf{s}^{(\ell)}[t] = \boldsymbol{\Theta}(\mathbf{u}^{(\ell)}[t] - \vartheta), \tag{2}$$

$$\mathbf{h}^{(\ell)}[t] = \mathbf{u}^{(\ell)}[t] \cdot (1 - \mathbf{s}^{(\ell)}[t]) + \mathbf{u}_{reset} \cdot \mathbf{s}^{(\ell)}[t] \tag{3}$$

where t respectively represent the indices of the time step and the l-th layer, $\mathbf{W}^{(\ell)} \in \mathbb{R}^{d \times d}$ denotes synaptic weight matrix between two adjacent layers, $f(\cdot)$ is the function operation which stands for convolution (Conv) or fully connected (FC), $\mathbf{x}^{(\ell-1)}[t-1] \in \mathbb{R}^{1 \times d}$ is the input, and $\boldsymbol{\Theta}(\cdot)$ denotes the Heaviside step function. When the membrane potential $\mathbf{u}^{(\ell)}[t]$ exceeds the firing threshold ϑ, the IF neuron will trigger a spike $\mathbf{s}^{(\ell)}[t]$. Moreover, $\mathbf{h}^{(\ell)}[t]$ represents the membrane potential after the trigger event which equals $\mathbf{u}^{(\ell)}[t]$ if no spike is generated and otherwise equals the reset potential $\mathbf{u}_{reset}$.

Flexibly adjustable integer spiking neuron Spiking neurons propagate information in both spatial and temporal domains, and they mimic the spiking communication scheme of biological neurons. However, there are inherent quantization errors in converting the membrane potential of spiking neurons into binary spikes, which severely limits the representational power of the model. Hence, we proposed the flexibly adjustable integer spiking neuron to reduce the quantization error. This neuron recognizes integer values with a single timestep while training and converts them into 0/1 spikes when inference. Mathematically, in training, Eq. 2 can be rewritten as:

$$\mathbf{s}^{(\ell)}[t] = \text{clip}\left(\lfloor \frac{\mathbf{u}^{(\ell)}[t]}{S} \rceil + Z, a, b\right) \tag{4}$$

where $\lfloor \cdot \rceil$ denotes the rounding operator, and clip$\{x, a, b\}$ confines x within quantization range $[a, b]$, with a and b representing the minimum and maximum quantized integer values, respectively. To simulate the integer spiking neuron, we use an asymmetric quantization strategy, setting the minimum value a to 0 and the maximum value b to $2^B - 1$, where B is the number of quantization bits. The quantization parameters S and Z , with S as the scaling factor and Z as the zero point, together map the dynamic range of the membrane potential to the quantization value range, which are calculated as:

$$S = \frac{\max(\mathbf{u}^{(\ell)}[t]) - \min(\mathbf{u}^{(\ell)}[t])}{b - a} \quad Z = \lfloor a - \frac{\min(\mathbf{u}^{(\ell)}[t])}{S} \rceil \tag{5}$$

Once quantization is done, the dequantization process maps the discrete quantized values back to the original membrane potential range, enabling the learning of quantization errors. The dequantization formula is:

$$\mathbf{u}^{(\ell)}[t] = (\mathbf{s}^{(\ell)}[t] - Z) \times S \tag{6}$$

This equation reverses the quantization process by applying the scaling factor S and zero point Z to recover the original membrane potential.

BackPropagation process of FAI-LIF There exist two primary methods of training high-performance SNNs. One way is to discretize ANN into spike form through neuron equivalence[51,52], i.e., ANN-to-SNN conversion, but this requires a long simulation time step and increases the energy consumption. Instead, we have employed the direct training method[55] and applied surrogate gradient training. In this section, we introduced the training process of

SNN gradient descent and the parameter update method of our FAI-LIF. SNNs' parameters can be taught using gradient descent techniques, just like ANNs, after determining the derivative of the generation process. Moreover, the accumulated gradients of loss $\mathcal{L}$ with respect to weights $\mathbf{w}$ at layer ℓ can be calculated as:

$$\frac{\partial \mathcal{L}}{\partial \mathbf{W}^{(\ell)}} = \sum_{t=1}^{T} \frac{\partial \mathcal{L}}{\partial \mathbf{s}^{(\ell+1)}[t]} \frac{\partial \mathbf{s}^{(\ell+1)}[t]}{\partial \mathbf{u}^{(\ell+1)}[t]} \left(\frac{\partial \mathbf{u}^{(\ell+1)}[t]}{\partial \mathbf{W}(\ell)} + \sum_{\tau<t} \prod_{i=t-1}^{\tau} \left(\frac{\partial \mathbf{u}^{(\ell+1)}[i+1]}{\partial \mathbf{u}^{(\ell+1)}[i]} + \frac{\partial \mathbf{u}^{(\ell+1)}[i+1]}{\partial \mathbf{s}^{(\ell+1)}[i]} \frac{\partial \mathbf{s}^{(\ell+1)}[i]}{\partial \mathbf{u}^{(\ell+1)}[i]} \right) \frac{\partial \mathbf{u}^{(\ell+1)}[\tau]}{\partial \mathbf{W}^{\ell}} \right) \tag{7}$$

where $\mathbf{s}^{(\ell)}[t]$ and $\mathbf{u}^{(\ell)}[t]$ represent the binary and membrane potential of the neuron in layer ℓ at time t, respectively. Moreover, notice that $\frac{\partial \mathbf{s}^{(\ell)}[t]}{\partial \mathbf{u}^{(\ell)}[t]}$ is non-differentiable. For simplicity, we retain gradients only for neurons activated within the $[0, D]$ range, setting all others to zero.

Directly training the above FAI-LIF-based SNNs requires the use of backpropagation through time (BPTT)[55], resulting in a time complexity of $\mathcal{O}(NT)$, where N and T is the number of layers and time steps. This significantly increases both the training time and memory requirements. To mitigate this issue, we have set the pretraining time step to 1. In addition, our FAI-LIF will emit integer values while training, and convert them into 0/1 spikes by extending the virtual timesteps to ensure that the inference is spike-driven with only sparse addition.

Supplementary Note 2: Details of spike linear attention method

Transformers employing softmax attention[28] facilitate efficient parallel training but are constrained by their quadratic complexity with respect to sequence length. Linear attention mitigates this limitation by optimizing computational efficiency, reducing the complexity to $\mathcal{O}(Ld)$, where L is the length of the tokens and d is the dimensions. While preserving a degree of global dependency modeling, it markedly enhances computational efficiency, rendering it more suitable for processing long sequences and large-scale datasets. In the following paragraphs, we first elucidate the inherent limitations of conventional attention mechanisms. We then introduce our novel Spike Linear Attention, formulated within a unified framework of linear attention, to address these challenges.

Vanilla softamax self-Attention Standard Transformers utilize a softmax attention mechanism that takes an input sequence $\mathbf{X} \in \mathbb{R}^{L\times d}$, where L represents the sequence length and d denotes the hidden dimension, and computes the output $\mathbf{O} \in \mathbb{R}^{L\times d}$ through the following process:

$$\begin{aligned} &\mathbf{Q} = \mathbf{W}_Q\mathbf{X}, \mathbf{K} = \mathbf{W}_K\mathbf{X}, \mathbf{V} = \mathbf{W}_V\mathbf{X} \\ &\mathbf{O} = \text{softmax}\left(\frac{\mathbf{Q}\mathbf{K}^\top}{\sqrt{d}}\right)\mathbf{V} \end{aligned} \tag{8}$$

where $\mathbf{W}_Q, \mathbf{W}_K, \mathbf{W}_V \in \mathbb{R}^{d\times d}$ are learnable matrices. The time complexity of VSA is $\mathcal{O}(L^2 d)$. During inference Transformers should use the following recurrent form, the above Eq. 8 can be rewritten as:

$$\begin{aligned} &\mathbf{q}_t = \mathbf{W}_Q\mathbf{x}_t, \mathbf{k}_t = \mathbf{W}_K\mathbf{x}_t, \mathbf{v}_t = \mathbf{W}_V\mathbf{x}_t \\ &\mathbf{o}_t = \frac{\sum_{i=1}^{t}\exp(\mathbf{q}_t\mathbf{k}_i^\top)\mathbf{v}_i}{\sum_{i=1}^{t}\exp(\mathbf{q}_t\mathbf{k}_i^\top)} \end{aligned} \tag{9}$$

where the computation of $\mathbf{q}_t$, $\mathbf{k}_t$, and $\mathbf{v}_t$ is derived from the current token's representation $\mathbf{x}_t \in \mathbb{R}^{1\times d}$, and attention is subsequently performed over the growing set of keys $\{\mathbf{k}_1, \ldots, \mathbf{k}_t\}$ and values $\{\mathbf{v}_1, \ldots, \mathbf{v}_t\}$.

Unified linear attention In the context of Unified Linear Attention, we have replaced the term $\exp(\mathbf{q}_t\mathbf{k}_i^\top)$ in Eq. 9 with a kernel function $\phi(\mathbf{xy})$ with $\phi(\mathbf{xy}) = \phi(\mathbf{x})\phi(\mathbf{y})$, thereby transforming the attention mechanism into a more efficient form. This substitution significantly simplifies the calculation of $\mathbf{o}_t$ in the aforementioned Eq. 9, as it eliminates the need for the computationally expensive exponential operation. Consequently, the attention computation becomes more scalable and amenable to faster processing, particularly for long sequences. The updated formulation can then be rewritten as:

$$\mathbf{o}_t = \frac{\sum_{i=1}^{t}\phi(\mathbf{q}_t)\phi(\mathbf{k}_i)^\top\mathbf{v}_i}{\sum_{i=1}^{t}\phi(\mathbf{q}_t)\phi(\mathbf{k}_i)^\top} = \frac{\phi(\mathbf{q}_t)\sum_{i=1}^{t}\phi(\mathbf{k}_i)^\top\mathbf{v}_i}{\phi(\mathbf{q}_t)\sum_{i=1}^{t}\phi(\mathbf{k}_i)^\top} \tag{10}$$

Thus Eq. 10 can be rewritten as an RNN with recurrent states $\mathbf{H}_t \in \mathbb{R}^{d\times d}$ and $\mathbf{z}_t \in \mathbb{R}^{d}$:

$$\mathbf{H}_t = \mathbf{H}_{t-1} + \phi(\mathbf{k}_t)^\top\mathbf{v}_t,\ \mathbf{z}_t = \mathbf{z}_{t-1} + \phi(\mathbf{k}_t)^\top \tag{11}$$

$$\mathbf{o}_t = \frac{\phi(\mathbf{q}_t)\mathbf{H}_t}{\phi(\mathbf{q}_t)\mathbf{z}_t} \tag{12}$$

By changing the outer product operation between $\mathbf{k}_t$ and $\mathbf{v}_t$ to the Hadamard product and treating the value vector $\mathbf{v}_t$ at the current moment separately, we can get an RNN with one-dimensional recurrent states $\mathbf{H}_t \in \mathbb{R}^{d}$ and $\mathbf{z}_t \in \mathbb{R}^{d}$:

$$\mathbf{wkv}_t = \frac{\mathbf{H}_{t-1} + e^{u}\phi(\mathbf{k}_t)\mathbf{v}_t}{\mathbf{z}_{t-1} + e^{u}\phi(\mathbf{k}_t)} \tag{13}$$

$$\mathbf{H}_t = e^{-w}\mathbf{H}_{t-1} + \phi(\mathbf{k}_t)\mathbf{v}_t,\ \mathbf{z}_t = e^{-w}\mathbf{z}_{t-1} + \phi(\mathbf{k}_t) \tag{14}$$

This is the WKV operator in RWKV linear attention, where $\phi(\mathbf{k}_t) = e^{\mathbf{k}_t}$.

Connection between spiking neurons and linear attention The RWKV structure exhibits a fascinating connection to Leaky Integrate-and-Fire (LIF) neurons. In an LIF neuron model, when the membrane potential $\mathbf{u}^{(\ell)}[t]$ does not exceed the firing threshold, it is updated according to:

$$\mathbf{u}^{(\ell)}[t] = \beta\mathbf{u}^{(\ell)}[t-1] + f(\mathbf{x}_t) \tag{15}$$

If we set $\beta = e^{-w}$, and choose $f(\mathbf{x}_t) = \phi(\mathbf{k}_t)\mathbf{u}_t$ or $f(\mathbf{x}_t) = \phi(\mathbf{k}_t)$, this update rule becomes equivalent to the RWKV state update process in Eq. 14. This illustrates the deep connection between LIF neurons and the RWKV operator, where the RWKV architecture mirrors the state update dynamics of LIF neurons, with the membrane potential update being governed by a decay factor and an input-dependent term.

Moreover, the time-mixing block in RWKV uses key variables: $\mathbf{r}_t$ (receptance vector), $\mathbf{k}_t$ (key vector), $\mathbf{v}_t$ (value vector), w (time decay), u (current token weighting), and $\sigma(\mathbf{r}_t)$ (forget gate). From a neuromorphic perspective, the time decay parameter w in RWKV functions in a similar method of temporal information aggregation to the membrane time constant τ in LIF neurons, introducing a recency bias that prioritizes recent inputs and gradually forgets older ones. The receptance mechanism $\sigma(\mathbf{r}_t)$ mirrors adaptive thresholding in neurons, similar to homeostatic plasticity. The $\mathbf{wkv}_t$ computation integrates weighted inputs based on temporal distance, relevance, and content, with normalization ensuring proper scaling. The token shift mechanism resembles dendritic computation, enhancing sensitivity to specific temporal patterns.

Spike linear attention We construct a spiking linear attention mechanism based on the relative position encoding and key-value separation techniques of the RWKV architecture[22]. Both RWKV and spiking neural networks share fundamental computational characteristics that make their integration natural and effective. Both utilize one-dimensional hidden states ($1 \times d$), employ element-wise operations (Hadamard product) for state transitions, and process information recurrently. This architectural similarity allows RWKV's linear attention mechanism to be efficiently adapted to the spiking neuron paradigm, leveraging the strengths of both approaches while maintaining computational efficiency. Next, we will introduce the mathematical expression of Spike Linear Attention. First, the calculation of the spike attention score is as follows:

$$\mathbf{s}_c^{(\ell)}[t] = \mathbf{W}_c \cdot (\mu_c \mathbf{s}_x^{(\ell)}[t] + (1-\mu_c)\mathbf{s}_x^{(\ell)}[t-1]), c \in \{r, k, v\} \tag{16}$$

where $\mathbf{s}_{(\cdot)}^{(\ell)}[t]$ represents the spike trains generated by Eq. 4, which maps the input into spike trains. $\mathbf{s}_r^{(\ell)}[t]$ is the spike receptance vector, which acts as a gate for information flow. $\mathbf{s}_k^{(\ell)}[t]$ is the spike key vector, determining the relevance of information at time t. And $\mathbf{s}_v^{(\ell)}[t]$ is the spike value vector, carrying the content to be processed. $\mathbf{W}_c$ and μ_c are trainable parameters, varying depending on the attention type used. We then introduce how to calculate the intermediate hidden states $\mathbf{H}[t]$ and output spike trains $\mathbf{s}_o^{(\ell)}[t]$:

$$\mathbf{s}_{\mathbf{wkv}}^{(\ell)}[t] = \frac{\mathbf{H}_{t-1} + e^{u+\mathbf{s}_k^{(\ell)}[t]}\mathbf{s}_v^{(\ell)}[t]}{\mathbf{z}_{t-1} + e^{u+\mathbf{s}_k^{(\ell)}[t]}} \tag{17}$$

$$\mathbf{H}[t] = e^{-w}\mathbf{H}[t-1] + e^{\mathbf{s}_k^{(\ell)}[t]}\mathbf{s}_v^{(\ell)}[t] \quad \mathbf{z}_t = e^{-w}\mathbf{z}_{t-1} + e^{\mathbf{s}_k^{(\ell)}[t]} \tag{18}$$

$$\mathbf{s}_o^{(\ell)}[t] = \mathbf{W}_o \cdot (\sigma(\mathbf{s}_r^{(\ell)}[t]) \odot \mathbf{s}_{\mathbf{wkv}}^{(\ell)}[t]) \tag{19}$$

where u is a weighting parameter for the current token, w is the time decay parameter, and $\mathbf{s}_o^{(\ell)}[t]$ is the output spike trains at time step t, with $\odot$ representing the element-wise multiplication.

Supplementary Note 3: Details of Spike neuron tools method

Moving average In the process of quantizing the membrane potential into integer spikes, we apply a moving average smoothing process to smooth the data distribution and reduce the impact of extreme values. The specific formulas are as follows:

$$\max(\mathbf{u}^{(\ell)}[t]) = \alpha \cdot \max(\mathbf{u}^{(\ell)}[t-1]) + (1-\alpha) \cdot \max(\mathbf{u}^{(\ell)}[t]) \tag{20}$$

$$\min(\mathbf{u}^{(\ell)}[t]) = \alpha \cdot \min(\mathbf{u}^{(\ell)}[t-1]) + (1-\alpha) \cdot \min(\mathbf{u}^{(\ell)}[t]) \tag{21}$$

where α is the decay factor (usually close to 1, e.g. 0.9).

Layer-wise quantization sensitivity To evaluate the relationship between activation quantization sensitivity, the types of network layers, and their positions in the network, we designed a layer-wise sensitivity formula to measure the error between the quantized and non-quantized models. The formula is as follows:

$$E(\beta,\gamma) = -\frac{1}{N}\sum_{i=1}^{N}\sum_{c=1}^{C} p_c(X_i)\log(\hat{p}_c(X_i)) + \frac{1}{N}\sum_{i=1}^{N}\sum_{c=1}^{C} p_c(X_i)\log(\tilde{p}_{\beta,\gamma,c}(X_i)) \tag{22}$$

In this formula, N denotes the total number of samples, and C represents the number of classes. $p_c(X_i)$ is the true probability distribution for sample X_i belonging to class c. $\hat{p}_c(X_i)$ represents the predicted probability from the non-quantized model for the same sample and class. $\tilde{p}_{\beta,\gamma,c}(X_i)$ denotes the predicted probability from the quantized model, where β refers to the layer name and γ specifies the quantization method or precision.

Mixed-precision computing fusion To reduce the computational and memory overhead associated with single-precision training, we introduce an Automatic Mixed Precision (AMP) strategy into the quantization training process of NSLLM. However, integrating AMP with quantization presents several challenges, including potential accuracy degradation, numerical errors, gradient instability, and compatibility issues. To avoid the complexity of multiple rounds of quantization-aware training and effectively address these challenges, we incorporate a fake quantization module into the AMP workflow to capture the combined loss introduced by mixed-precision and quantization training. Furthermore, to mitigate gradient overflow, we adopt the BF16 format, which offers a wider numerical range compared to FP16, and employ more robust quantization methods to reduce numerical errors and enhance overall training stability. (See Figs. S3–S4 for the specific design and performance of our AMP module.)

Quantile sparse quantization To assist sparsity by modifying the distribution shape of the membrane potential data, we have designed a quantile-shifted correction linear unit before quantization. First, the quantile X_q for a given quantile ratio q is calculated as

$$X_q = \text{quantile}(X, q) \tag{23}$$

where X represents the input data, and q is the quantile ratio. Based on this, the ReLU function is applied to achieve sparsification as

$$X_{\text{relu}} = \text{ReLU}(X - X_q) \tag{24}$$

After sparsification, the model undergoes a quantization process. We rewrite X_{relu} as $\mathbf{u}^{\ell}_{\text{relu}}[t]$, and the quantization formula Eq. 4 is rewritten as:

$$\mathbf{s}^{(\ell)}[t] = \text{clip}\left(\lfloor \frac{\mathbf{u}^{\ell}_{\text{relu}}[t]}{S} \rceil + Z, a, b\right) \tag{25}$$

Virtual timesteps unfolding During inference, we refer to the approach from our previous work[56], the integer spike value $\mathbf{s}^{(\ell)}[t]$ is converted into a binary spike sequence $\mathbf{s}^{(\ell)}[t,d]$, where each spike value is either 0 or 1, and the total sum of the binary spikes equals $\mathbf{s}^{(\ell)}[t]$. To achieve this, the time step T is extended to $T \times D$, and the integer spike $\mathbf{s}^{(\ell)}[t]$ is decomposed into a spike sequence of length D. The input to the neuron at the $\ell+1$ layer can then be expressed as the weighted sum of the spike sequence using the weight matrix $\mathbf{W}^{(\ell+1)}$. By leveraging the linear property of matrix multiplication, the input $\mathbf{u}^{(\ell+1)}[t]$ is computed as:

$$\mathbf{u}^{(\ell+1)}[t] = \sum_{d=1}^{D}(\mathbf{W}^{(\ell+1)}\mathbf{s}^{(\ell)}[t,d]) \tag{26}$$

Operation counts evaluation In this section, we first calculate the spiking version of FLOPs for the NSLLM as follows:

$$\mathrm{FLOPs}^{(\ell)} = T \times fr^{(\ell)} \times \mathrm{FLOPs}^{(\ell)} \tag{27}$$

where T represents the number of time steps, and $fr^{(\ell)}$ is the firing rate at layer ℓ. It is important to note that the memory usage of quantized values differs from that of floating-point values[57]. We calculate the floating-point operations equivalent to integer operations by scaling the floating-point operations according to the memory usage ratio for different quantization bit-widths[57]. For instance, 2-bit multiplications are equivalent to $\frac{1}{32}$ floating-point operations in terms of FLOPs[58,59]. A 2-bit by 4-bit multiplication can be considered as two 2-bit multiplications. We present the FLOPs calculation formulas for different layer types in LLM (non-spiking state) and NSLLM (spiking state) in Table S3. In Table S4, we compare the FLOPs and key parameters for LLM and NSLLM.

Supplementary Note 4: Different layer bits and FLOPs of NSLLM

Table S1. The NSLLM model with 0.169B parameters uses a mixed-time-step binary spike configuration. Based on layer-wise quantization sensitivity, we configured the specific time steps for each layer and averaged the overall time-step count based on computational load. (We restricted the precision of LayerNorm, ElementWise Mul, and interpolation operators to integers, as their FLOPs are 3 to 4 orders of magnitude smaller than the Linear layers, so they are not included in the display.)

Layer Name	Layers	Sequence Length	Input Dimensions	Output Dimensions	Time Step
ATT.Key	12	1024	768	768	15
ATT.Value	12	1024	768	768	31
ATT.Receptance	12	1024	768	768	15
ATT.Output	12	1024	768	768	15
FFN.Key	12	1024	768	768	31
FFN.Value	12	1024	768	768	15
FFN.Receptance	12	1024	768	768	15
Head	12	1024	768	768	31
AVG			24		

Table S2. The NSLLM model with 1.5B parameters uses a mixed-time-step binary spike configuration. Based on layer-wise quantization sensitivity, we configured the specific time steps for each layer and averaged the overall time-step count based on computational load. (We restricted the precision of LayerNorm, ElementWise Mul, and interpolation operators to integers, as their FLOPs are 3 to 4 orders of magnitude smaller than the Linear layers, so they are not included in the display.)

Layer Name	Layers	Sequence Length	Input Dimensions	Output Dimensions	Time Step
ATT.Key	24	1024	2048	2048	63
ATT.Value	24	1024	2048	2048	15
ATT.Receptance	24	1024	2048	2048	31
ATT.Output	24	1024	2048	2048	15
FFN.Key	24	1024	2048	2048	15
FFN.Value	24	1024	2048	2048	15
FFN.Receptance	24	1024	2048	2048	15
Head	24	1024	2048	2048	63
AVG			23		

Table S3. FLOPs calculation formulas for different layer types in LLM and NSLLM. N is the sequence length, D is the embedding dimension (with D_I as the input embedding dimension and D_O as the output embedding dimension of the linear layer), T is the number of time steps, R is the spiking rate, k is a constant representing a single-digit integer, and ρ represents the ratio of FLOPs for integer operations to FLOPs for floating-point operations. The linear layer dominates the overall FLOPs, with its computational cost exceeding that of other layers by several orders of magnitude. Linear layers are implemented without bias; even if a bias term were included, its cost would be negligible compared to matrix multiplication.

LayerName	LLM	NSLLM
Linear	$N \cdot D_I \cdot D_O$	$T \cdot R \cdot N \cdot D_I \cdot D_O \cdot \rho$
LayerNorm	$N \cdot D \cdot k$	$T \cdot R \cdot N \cdot D \cdot k \cdot \rho$
Element-wise Mul	$N \cdot D \cdot k$	$T \cdot R \cdot N \cdot D \cdot k \cdot \rho$
Interpolation	$N \cdot D \cdot k$	$T \cdot R \cdot N \cdot D \cdot k \cdot \rho$

Table S4. Comparison of FLOPs and key parameters between LLM and NSLLM. We represent 1/32 FLOP as the equivalent 2-bit integer FLOPs, where operations involving 4-bit weights and 2-bit activations are equivalent to Two 2-bit integer FLOPs. (Layer spiking firing rate details can be found in Tables S5-S8.)

Model	Params (B)	Time Step	Bits	Firing Rate	FLOPs (G)
RWKV-4	0.169	-	32-32	-	133.76
NSLLM	0.169	24	4-1	0.3807	76.38
NSLLM*	0.169	24	4-1	0.1355	27.19
RWKV-4	1.5	-	32-32	-	1445.47
NSLLM	1.5	23	4-1	0.3027	628.97
NSLLM*	1.5	23	4-1	0.0504	104.72

Supplementary Note 5: Details of layer spiking firing rate

Table S5. Layer spiking firing rate details of NSLLM (0.169B, 4-1) on multiple zero-shot benchmark tasks.

LayerName	Winogrande	ARC-E	ARC-C	HeadQA	OpenBookQA	PIQA
ATT.Key	0.3273	0.3270	0.3240	0.3217	0.3323	0.3265
ATT.Value	0.3374	0.3370	0.3341	0.3309	0.3420	0.3368
ATT.Receptance	0.3260	0.3258	0.3230	0.3207	0.3292	0.3250
ATT.Output	0.5016	0.4979	0.4985	0.4847	0.4958	0.4953
FFN.Key	0.4356	0.4365	0.4356	0.4632	0.4451	0.4364
FFN.Value	0.4462	0.4493	0.4487	0.4713	0.4526	0.4472
FFN.Receptance	0.0064	0.0063	0.0063	0.0053	0.0063	0.0064
Head	0.6101	0.6069	0.6025	0.6276	0.5924	0.5866

Table S6. Layer spiking firing rate details of NSLLM* (0.169B, 4-1) on multiple zero-shot benchmark tasks.

LayerName	Winogrande	ARC-E	ARC-C	HeadQA	OpenBookQA	PIQA
ATT.Key	0.1505	0.1476	0.1457	0.1430	0.1648	0.1475
ATT.Value	0.1374	0.1360	0.1351	0.1359	0.1472	0.1355
ATT.Receptance	0.1235	0.1254	0.1258	0.1304	0.1263	0.1249
ATT.Output	0.0365	0.0386	0.0395	0.0311	0.0338	0.0394
FFN.Key	0.1977	0.1962	0.1952	0.1944	0.2142	0.1935
FFN.Value	0.1746	0.1787	0.1796	0.1827	0.1757	0.1761
FFN.Receptance	0.0065	0.0064	0.0064	0.0056	0.0065	0.0065
Head	0.1830	0.2020	0.2034	0.1755	0.1954	0.2000

Table S7. Layer spiking firing rate details of NSLLM (1.5B, 4-1) on multiple zero-shot benchmark tasks.

LayerName	Winogrande	ARC-E	ARC-C	HeadQA	OpenBookQA	PIQA
ATT.Key	0.3023	0.3021	0.3024	0.2885	0.2984	0.3054
ATT.Value	0.3238	0.3225	0.3228	0.3077	0.3224	0.3253
ATT.Receptance	0.3012	0.3035	0.3039	0.2872	0.2946	0.3061
ATT.Output	0.5158	0.5150	0.5153	0.5221	0.5192	0.5168
FFN.Key	0.4180	0.4292	0.4305	0.4209	0.4363	0.4360
FFN.Value	0.4213	0.4335	0.4353	0.4249	0.4368	0.4396
FFN.Receptance	0.0023	0.0022	0.0022	0.0018	0.0022	0.0021
Head	0.6703	0.6504	0.6537	0.5897	0.6376	0.6322

Table S8. Layer spiking firing rate details of NSLLM* (1.5B, 4-1) on multiple zero-shot benchmark tasks.

LayerName	Winogrande	ARC-E	ARC-C	HeadQA	OpenBookQA	PIQA
ATT.Key	0.0392	0.0407	0.0409	0.0426	0.0383	0.0412
ATT.Value	0.0372	0.0389	0.0391	0.0412	0.0368	0.0395
ATT.Receptance	0.0386	0.0403	0.0404	0.0419	0.0383	0.0407
ATT.Output	0.0117	0.0131	0.0134	0.0116	0.0110	0.0133
FFN.Key	0.0928	0.1023	0.1024	0.1086	0.1001	0.1034
FFN.Value	0.0901	0.0988	0.0989	0.1044	0.0974	0.1002
FFN.Receptance	0.0019	0.0019	0.0019	0.0016	0.0019	0.0019
Head	0.0609	0.0646	0.0653	0.0480	0.0640	0.0636

Supplementary Note 6: Sensitivity analysis of layer-wise quantization errors

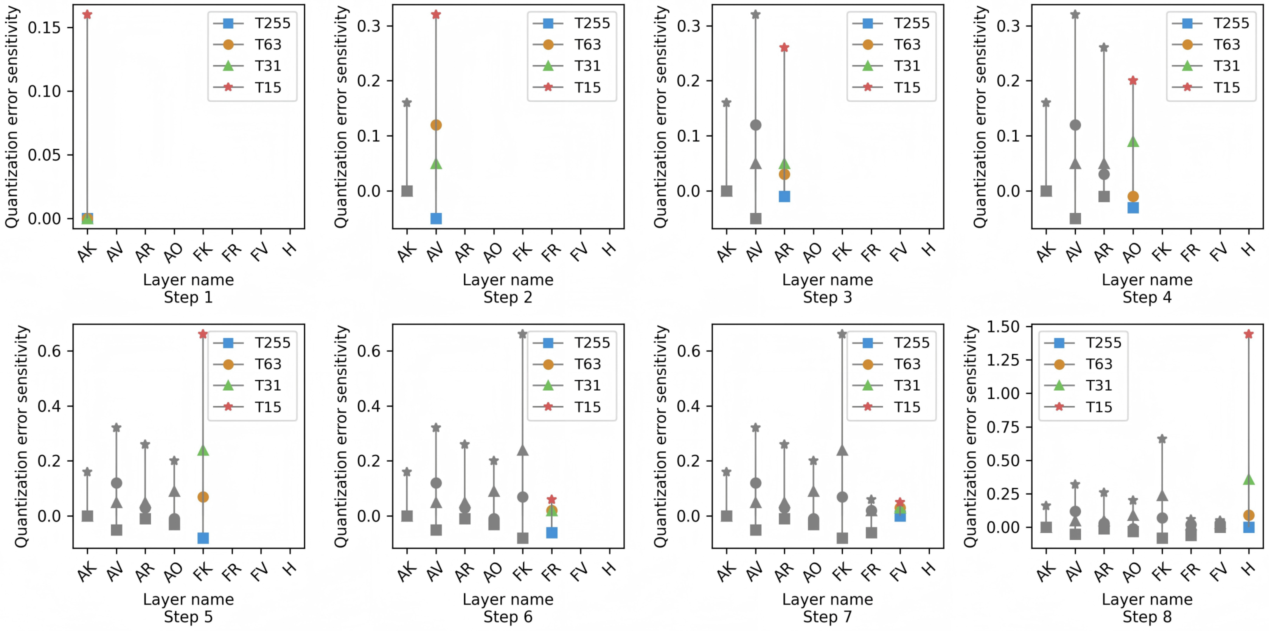

Figure S1. Layer-wise quantization and performance analysis algorithm. The layer-wise quantization error sensitivity analysis process for the 0.169B model with spiking neurons under different quantization modes (A1T15, A1T31, A1T64, A1T255), each converted respectively from A4T1, A5T1, A6T1, and A8T1 during training.

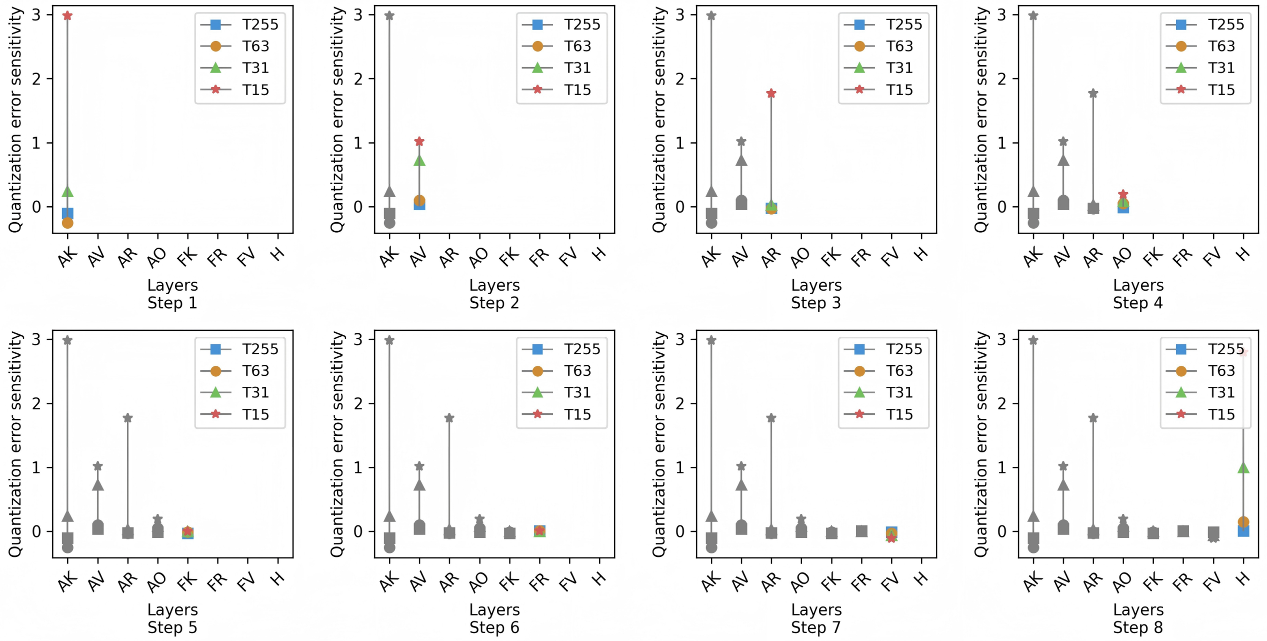

Figure S2. Layer-wise quantization and performance analysis algorithm. The layer-wise quantization error sensitivity analysis process for the 1.5B model with integer spiking neurons under different quantization modes (A1T15, A1T31, A1T64, A1T255), each converted respectively from A4T1, A5T1, A6T1, and A8T1 during training.

Supplementary Note 7: Performance comparison of different training precision formats

To avoid the complexity of multiple quantization-aware training sessions and effectively address the potential challenges of combining quantization with mixed precision, we integrated the Automatic Mixed Precision (AMP) strategy into NSLLM and adopted more robust quantization training methods. As shown in Fig. S3, in our quantized AMP strategy, the forward pass uses the Observer module with a moving average operation to determine the dynamic range of the input data and quantizes the 16-bit floating-point numbers (16-bit) into lower-bit integer representations (q-bit) to simulate low-precision computation. Then, these quantized data are dequantized back to 16-bit floating-point numbers to ensure the numerical stability for subsequent computations. The entire backward pass is also performed in 16-bit floating-point precision to reduce memory usage and increase computational speed. During the update phase of the model parameters, the optimizer calculates gradient updates in FP16 precision with the model weights (i.e., the optimizer's parameters) still stored in 32-bit floating-point (FP32) format. Through this process, gradient clipping techniques can be combined to clip gradient values before updating parameters to prevent gradient explosion. To ensure the stability and accuracy of the training process, the AMP strategy uses GradScaler to scale gradients and updates model weights with clipped gradients. By combining quantization training with the Automatic Mixed Precision (AMP) strategy and gradient clipping techniques, AMP effectively balances computational efficiency and model performance, making it suitable for the high-efficiency computing needs in deep learning.

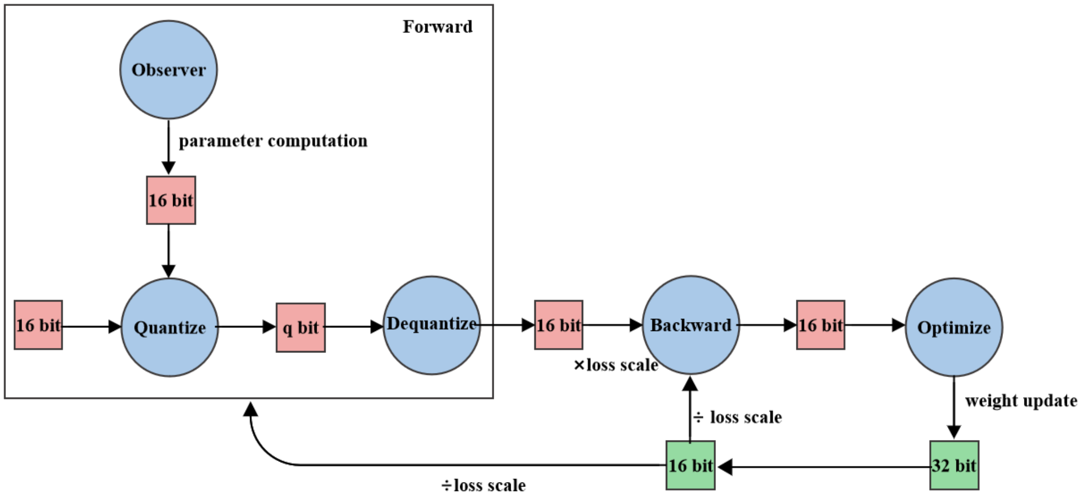

Figure S3. AMP process for integrating quantization training. The process includes the observer for parameter computation, the quantization and dequantization steps, loss scaling, and the forward and backward propagation for weight updates.

We evaluated the performance of mixed-precision computation across different batch sizes by testing a 169M parameter model on an A800 GPU with a fixed sequence length of 1024. In the test experiments, we chose BF16 as the 16-bit precision type and compared it with FP32 and TF32 (both 32-bit precision types). As shown in Fig. S4a, BF16 and TF32 have very similar GPU computation times (GPU Time), but there is a significant difference compared to FP32. In terms of GPU memory usage (GPU Memory), the difference between BF16 and both FP32 and TF32 is substantial and increases as the batch size grows. Due to experimental limitations, the largest batch size we tested was 256. At this batch size, BF16 used only 60% of the GPU memory compared to FP32 and TF32, and the speedup was 2.6 times that of FP32. Fig. S4b shows the loss curves and training times for these three models, demonstrating that the BF16 model converges to a similar loss as the 32-bit models in a shorter time. Fig. S4c compares the performance of BF16, TF32, and FP32 across multiple benchmark tasks, showing that BF16 performs almost identically to FP32 and TF32.

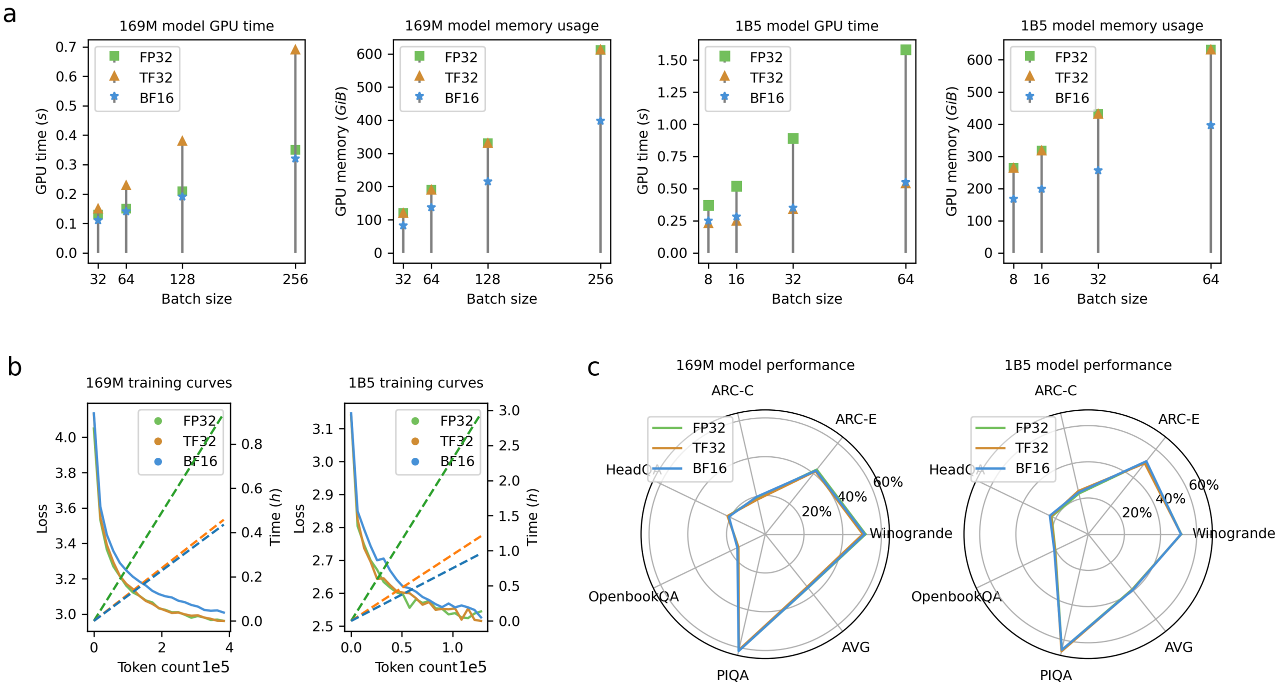

Figure S4. Performance comparison of different training precision formats. **(a)** Comparison of GPU time and memory usage for BF16, TF32, and FP32 training precision formats. **(b)** Comparison of the correlation between loss and training time for BF16, TF32, and FP32 training precision formats, with the loss curve on the left and the training time curve on the right. **(c)** Comparison of the performance of BF16, TF32, and FP32 training precision formats across multiple benchmark tasks.

Supplementary Note 8: Visualization of spike firing distributions across different layers

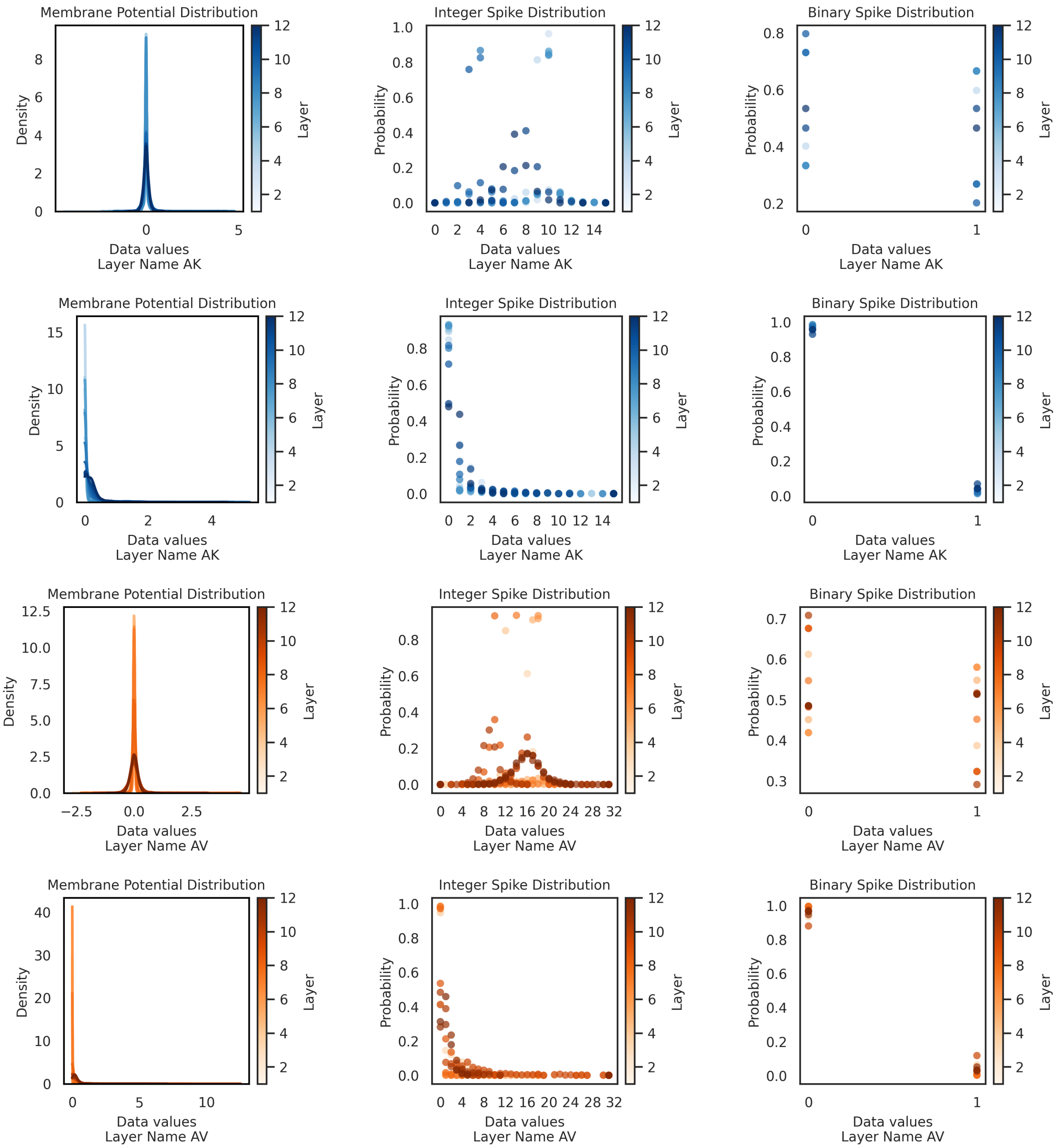

Figure S5. Visualization of spike firing distributions in the AK layer (top two rows) and AV layer (bottom two rows) for the spike model (NSLLM) and sparse spike model (NSLLM*). The first row of each layer corresponds to NSLLM and the second row to NSLLM*. The left column shows the membrane potential distribution, the middle column shows the integer spike distribution, and the right column shows the binary spike distribution. The color gradient represents the depth of different layers. The model configuration is based on 169M, 4-bit integer weights and mixed-time-step binary spikes.

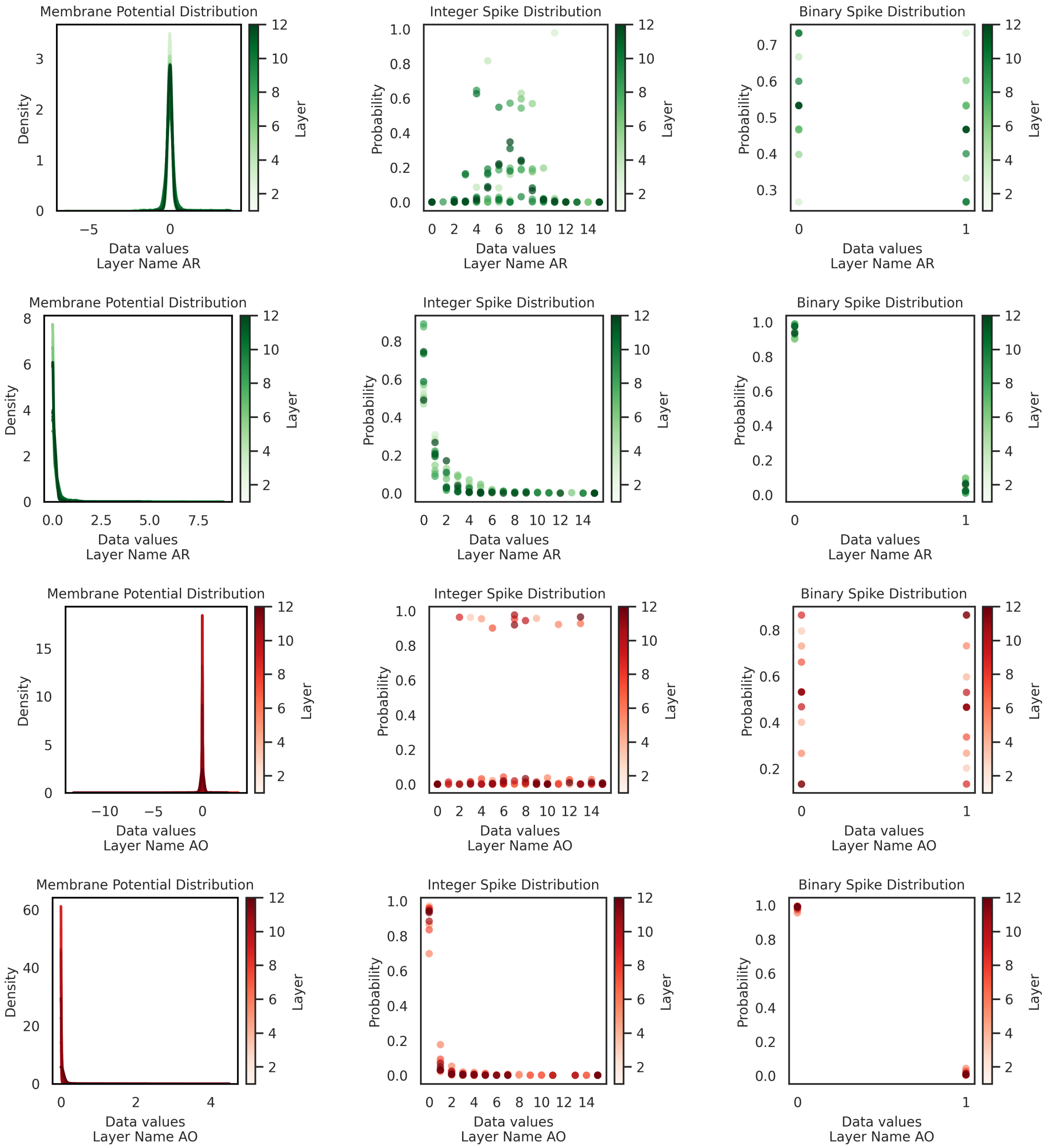

Figure S6. Visualization of spike firing distributions in the AR layer (top two rows) and AO layer (bottom two rows) for the spike model (NSLLM) and sparse spike model (NSLLM*). The first row of each layer corresponds to NSLLM and the second row to NSLLM*. The left column shows the membrane potential distribution, the middle column shows the integer spike distribution, and the right column shows the binary spike distribution. The color gradient represents the depth of different layers. The model configuration is based on 169M, 4-bit integer weights and mixed-time-step binary spikes.

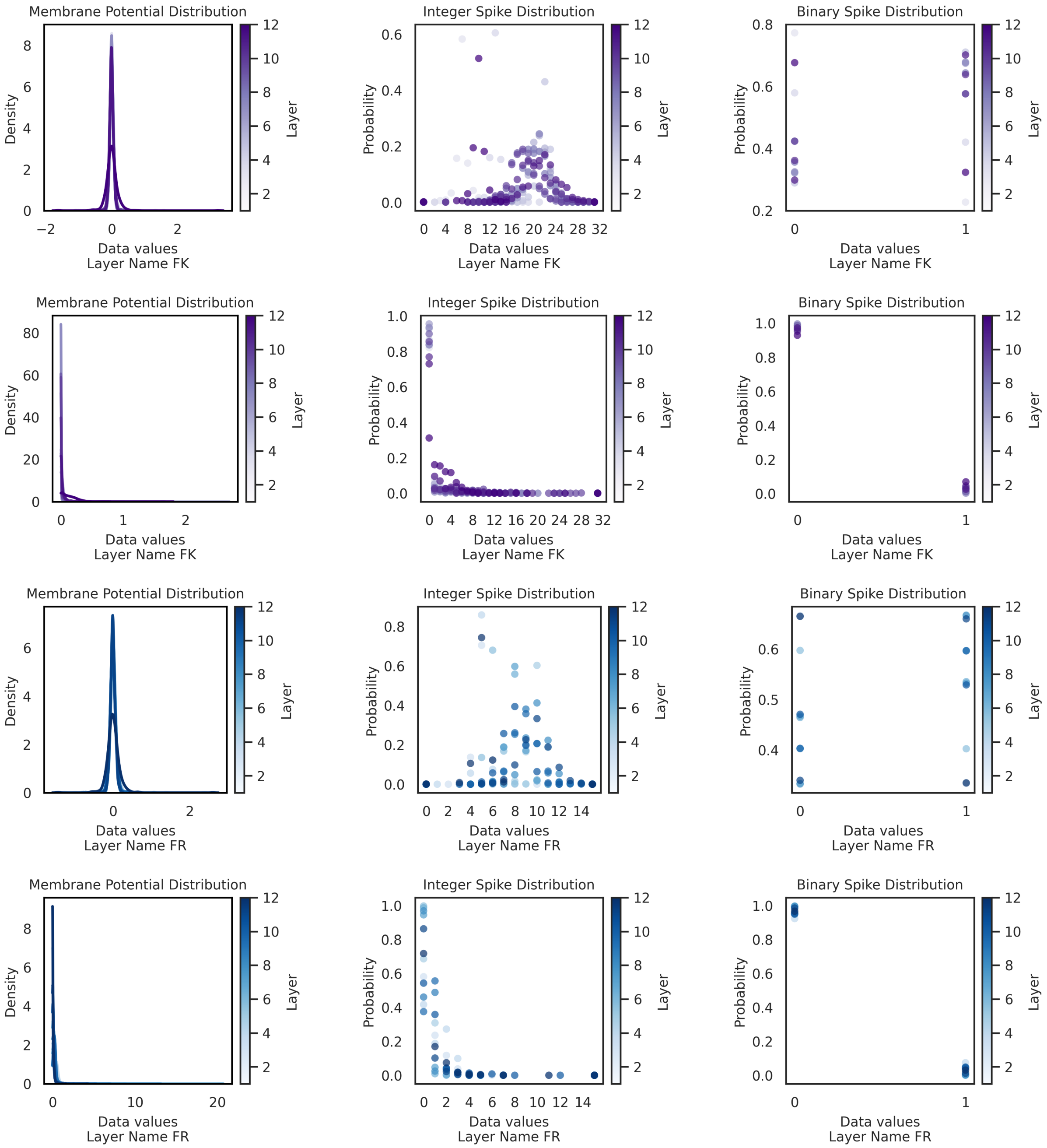

Figure S7. Visualization of spike firing distributions in the FK layer (top two rows) and FR layer (bottom two rows) for the spike model (NSLLM) and sparse spike model (NSLLM*). The first row of each layer corresponds to NSLLM and the second row to NSLLM*. The left column shows the membrane potential distribution, the middle column shows the integer spike distribution, and the right column shows the binary spike distribution. The color gradient represents the depth of different layers. The model configuration is based on 169M, 4-bit integer weights and mixed-time-step binary spikes.

871 ## Supplementary Note 9: Details of spike encoding

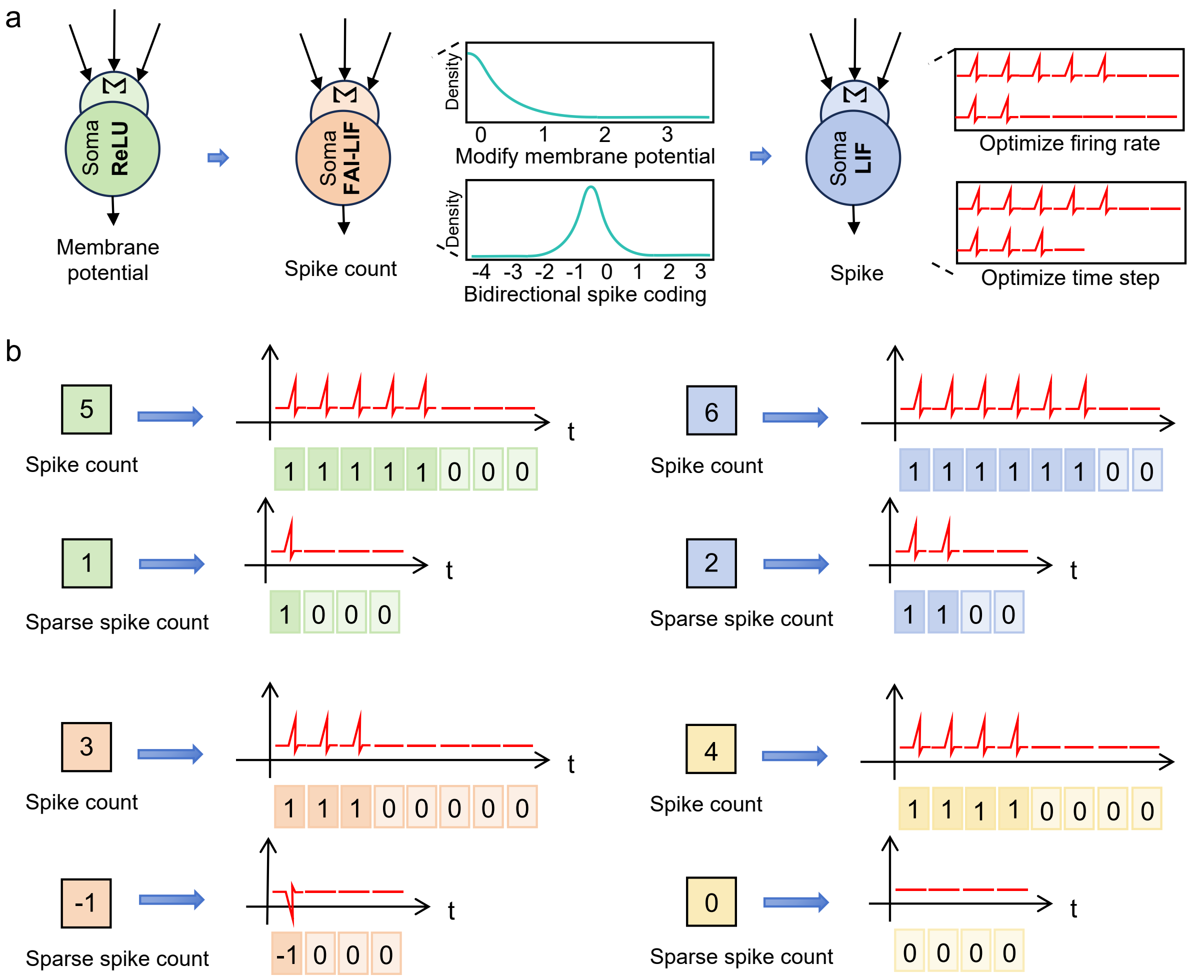

Figure S8. Schematic of spike encoding for the spike model (NSLLM) and sparse spike model (NSLLM*). (a) The directional sparse optimization technique maps membrane potentials into spike counts, which are then unfolded into sparse spike trains across virtual time steps. This achieves an efficient conversion and optimization from continuous activations to discrete spikes. Meanwhile, by optimizing the firing rate and time step, the energy efficiency of spike encoding is further enhanced. (b) Illustration of two directional sparse optimization methods. The left panel shows bidirectional encoding, which reconstructs the mapping from membrane potential to spike count so that low-amplitude counts occupy a larger probability mass statistically, thereby effectively absorbing the high-frequency large-count values from the tail of the original distribution. For example, when the spike count is 5, the traditional encoding is represented as "1111100," whereas the sparse spike count is expressed as "1000" with a much lower firing rate while keeping the time steps unchanged. The right panel shows the membrane potential partitioning method, which also ensures that low-amplitude counts occupy a larger probability mass, with an even greater proportion of zero counts. For instance, when the spike count is less than 4, the sparse spikes are all encoded as "0000," and the output contains only 0/1 spike representations.

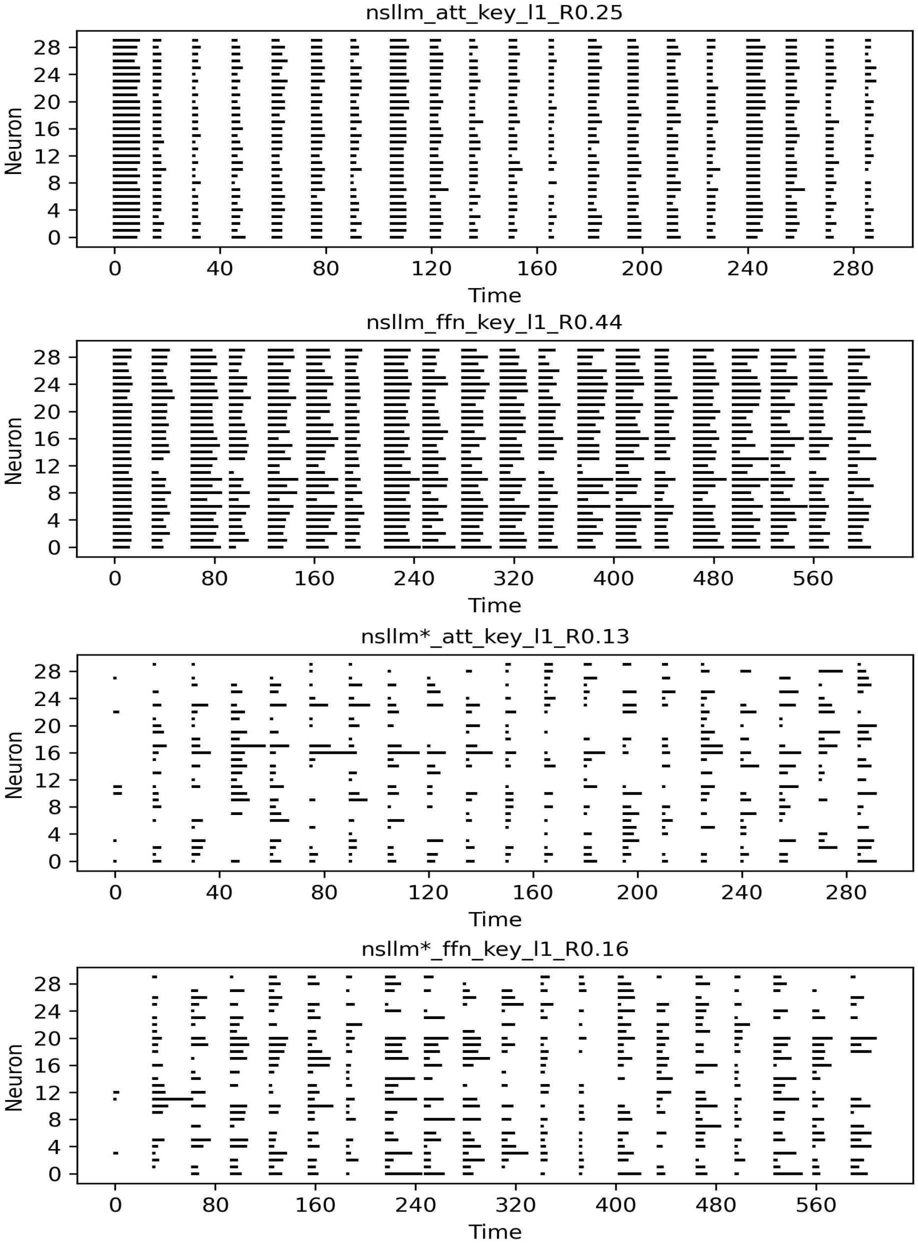

Figure S9. Spike firing visualization of the spike model (NSLLM) and sparse spike model (NSLLM*). The horizontal axis represents Time, corresponding to the total duration of token time steps × extended time steps; the vertical axis represents Neuron indices. Each black dot indicates a spike firing event of the corresponding neuron at that time point. Compared with the spike model, the sparse spike model exhibits significantly sparser spike firing patterns.

Supplementary Note 10: Supplementary results for Table 1

In the main text, we primarily present the performance of our model on a variety of zero-shot benchmark tasks (see Table 1). Furthermore, as shown in Table S9, we additionally evaluate NSLLM on more challenging benchmarks, including GSM8K[60] (mathematical reasoning), SQuAD[61] (reading comprehension), and TriviaQA[21] (world knowledge). The results demonstrate that even on these complex tasks, our model maintains high accuracy and robustness, confirming the generality and effectiveness of the proposed approach.

Table S9. Evaluation of NSLLM on more challenging benchmarks, including GSM8K (math, 8-shot), SQuAD (reading comprehension, zero-shot), and TriviaQA (world knowledge, 5-shot). 'Bits' denotes the bit width of the weight and activity, respectively. * indicates the use of a spiking sparsity strategy. For GSM8K, we report *strict-match (Str.)* and *flexible-extract (Flex.)* accuracy. For SQuAD, we report *Exact Match (EM)*, *F1*, *HasAns_Exact (HA-EM)*, *HasAns_F1 (HA-F1)*, and *NoAns_F1 (NA-F1)*. For TriviaQA , we report *Exact Match (EM)*.

Architecture	Bit	Spike -driven	Time Step	Params (B)	Flops (T)	GSM8K		SQuAD					TriviaQA
						Str.	Flex.	EM	F1	HA-EM	HA-F1	NA-F1	EM
LLAMA-2	16-16	×	N/A	7	6.91	15.30	15.30	16.77	24.21	24.68	39.37	8.66	64.14
**NSLLM* (Ours)**	**4-1.5**	✓	**8**	**7**	**0.75**	**6.36**	**6.67**	**19.16**	**25.52**	**18.96**	**31.52**	**19.37**	**51.71**
**NSLLM* (Ours)**	**4-1.5**	✓	**16**	**7**	**1.53**	**10.00**	**10.15**	**13.88**	**22.53**	**20.76**	**37.86**	**6.82**	**58.86**
**NSLLM* (Ours)**	**6-1.5**	✓	**32**	**7**	**4.60**	**14.85**	**1.39**	**18.05**	**25.12**	**24.82**	**38.77**	**11.12**	**64.05**
LLAMA-2	16-16	×	N/A	13	13.42	22.73	22.88	22.77	29.67	37.19	50.82	7.98	70.45
**NSLLM* (Ours)**	**4-1.5**	✓	**8**	**13**	**1.40**	**15.91**	**16.21**	**21.29**	**28.49**	**37.52**	**51.75**	**4.64**	**62.44**
**NSLLM* (Ours)**	**4-1.5**	✓	**16**	**13**	**2.89**	**21.06**	**21.36**	**21.15**	**28.82**	**33.07**	**48.21**	**8.94**	**66.45**
**NSLLM* (Ours)**	**6-1.5**	✓	**32**	**13**	**8.74**	**21.52**	**21.82**	**21.99**	**29.06**	**34.46**	**48.43**	**9.21**	**69.84**
LLAMA-3	16-16	×	N/A	8	7.97	48.64	49.55	26.71	32.58	52.69	64.30	0.07	71.58
**NSLLM* (Ours)**	**4-1.5**	✓	**8**	**8**	**0.84**	**18.80**	**19.41**	**19.65**	**27.43**	**37.66**	**52.59**	**0.05**	**49.31**
**NSLLM* (Ours)**	**4-1.5**	✓	**16**	**8**	**1.68**	**31.84**	**32.60**	**24.55**	**30.89**	**48.37**	**60.89**	**0.14**	**60.40**
**NSLLM* (Ours)**	**6-1.5**	✓	**32**	**8**	**5.09**	**46.55**	**47.16**	**28.32**	**34.74**	**54.31**	**66.61**	**0.05**	**70.26**

Supplementary Note 11: Optimized algorithm-hardware co-design with ternary weights for MatMul-free multiplication

In modern computational systems, hardware-unfriendly operations often emerge as critical performance bottlenecks. To address this challenge, we introduced the NSLLM framework. We select ternary weights of $-1, 0, +1$ and meticulously orchestrate the data flow between weights and activations, ensuring that the system, when deployed on hardware, transforms into a non-matrix multiplication unit. Specifically, the matrix multiplication operations within the model are eliminated, leaving only additive operations. The following describes the specific procedure: In a standard fully connected layer, the matrix multiplication (MatMul) between the input vector $\mathbf{x} \in \mathbb{R}^{1\times d}$ and the weight matrix $\mathbf{W} \in \mathbb{R}^{d\times m}$ is expressed as:

$$\mathbf{y} = \mathbf{W}\mathbf{x} = \sum_{k=1}^{d} w_{jk} x_k \quad \text{for } j = 1, 2, \ldots, n \tag{28}$$

where $\mathbf{y} \in \mathbb{R}^{1\times n}$ denotes the output. To circumvent the conventional dense fully connected layers, we introduce a MatMul-free unit that employs ternary weights to transform matrix multiplication operations into straightforward additive processes (i.e., ternary accumulation). In this framework, the elements of the weight matrix $\mathbf{W}$ are constrained to the ternary set $\{-1, 0, +1\}$. Let $\mathbf{W}^{''}$ represent the ternary weight matrix; the matrix multiplication operation with ternary weights is subsequently expressed as:

$$\mathbf{y}^{''} = \mathbf{x} \otimes \mathbf{W}^{''} = \sum_{j=1}^{d} x_j w^{''}_{jk}, \quad w^{''}{}_{jk} \in \{-1, 0, +1\}, \quad \text{for } j = 1, 2, \ldots, n \tag{29}$$

where $\mathbf{y}^{''} \in \mathbb{R}^{1\times m}$ represents the output, and the symbol $\otimes$ indicates ternary matrix multiplication, which simplifies to an accumulation operation. Since the ternary weights $w^{''}_{jk}$ can only take values from $\{-1, 0, +1\}$, the multiplication within the MatMul operation can be replaced by straightforward addition or subtraction. The ternary matrix multiplication can then be expressed as:

$$y^{''}_k = \sum_{j=1}^{d} x_j w^{''}_{jk} = \sum_{j\in\{w^{''}_{jk}=1\}} x_j - \sum_{j\in\{w^{''}_{jk}=-1\}} x_j, \quad \text{for } j = 1, 2, \ldots, n \tag{30}$$

In this way, we replaced the conventional matrix multiplication with a simplified addition operation, which both circumvents hardware-unfriendly matrix multiplications and optimizes the solution for hardware deployment.

Supplementary Note 12: RWKV as a MatMul-free language model

Recent work has demonstrated that MatMul operations can be entirely eliminated from language models while maintaining strong performance[47]. Building on these insights, we observe that the RWKV architecture can be naturally adapted into a MatMul-free language model, as it already relies primarily on element-wise operations rather than matrix multiplications.

The RWKV architecture implements token mixing through a linear recurrence pattern that can be computed using only element-wise operations. The key operations in RWKV's token mixer can be expressed as:

$$\begin{aligned}
\mathbf{k}_t &= (\mu_k \mathbf{x}_t + (1-\mu_k)\mathbf{x}_{t-1}) \circledast \mathbf{W}_k \\
\mathbf{v}_t &= (\mu_v \mathbf{x}_t + (1-\mu_v)\mathbf{x}_{t-1}) \circledast \mathbf{W}_v \\
\mathbf{h}_t &= \frac{\mathbf{a}_{t-1} + e^{\mathbf{m}+\mathbf{k}_t} \odot \mathbf{v}_t}{\mathbf{b}_{t-1} + e^{\mathbf{m}+\mathbf{k}_t}}
\end{aligned} \tag{31}$$

where $\mathbf{W}_k$ and $\mathbf{W}_v$ are weight matrices that can be quantized to ternary values $\{-1, 0, 1\}$, $\circledast$ represents ternary accumulation, and $\odot$ denotes element-wise multiplication. The mixing coefficients μ_k and μ_v control the temporal blending of current and previous states.

While RWKV is MatMul-free, its practical implementation needs careful handling of exponential and division operations in its time-mixing mechanism. These can be approximated using lookup tables or piece-wise linear functions for hardware efficiency, or managed in log space for numerical stability. The dual hidden states $\mathbf{a}_t$ and $\mathbf{b}_t$ double the hidden dimension, requiring more memory. Despite these challenges, RWKV represents a promising direction for MatMul-free language modeling, offering efficient sequential processing without the quadratic complexity of attention mechanisms.

Supplementary Note 13: Details of FPGA implementation

To evaluate the energy consumption and efficacy of the NSLLM on bespoke hardware optimized for ternary operations, we developed an accelerator architecture and implemented it on FPGA. An overview of this design is presented in Fig. 5, which depicts the implementation of the FPGA accelerator tailored for the NSLLM. This accelerator is specifically engineered to enhance power efficiency and performance on custom hardware capable of fully exploiting ternary operations. It incorporates three key functional units: CPU, AI Engine, MatMul-free unit, each supporting seamless out-of-order execution.

Central to the accelerator's architecture are several crucial components: the Program Counter, responsible for tracking the sequence of instruction execution; the Instruction ROM, which houses the instruction set; the Register Router, which allocates incoming instructions to available registers; and the Register File, consisting of eight registers, each storing a vector in a distinct SRAM array. Each SRAM array is equipped with both read and write ports, with each port being dedicated to a single instruction at a time. When an instruction attempts to access a busy functional unit or register, the Program Counter induces a stall, delaying execution until the unit or register becomes available. Instructions that do not conflict are executed concurrently.

The Root Mean Square functional unit preserves numerical precision through a specialized hardware algorithm, operating in three distinct stages. In Stage 1, the target vector is copied into an internal temporary register, and each element is squared using a lookup table. Stage 2 applies a divide-and-conquer approach to average neighboring elements of the vector, thereby computing the Root Mean Square result. Finally, Stage 3 normalizes the original vector by dividing each element by the computed Root Mean Square. This divide-and-conquer methodology substantially reduces rounding errors when compared to conventional rolling sum and large division techniques.

The ternary MatMul-free unit is designed to perform the Matmul-free operation on a specified vector, with the corresponding ternary matrix accessed from DRAM. In our architecture, all ternary matrices are stored in DRAM. During the Matmul-free operation, an SRAM FIFO is populated with sequential fetches from DRAM, and then drained via an energy-efficient ternary addition operation. Presently, the three Matmul-free instructions account for the majority of the total execution time. Future iterations of this design will introduce parallelism and caching strategies to further optimize Matmul-free execution efficiency.

Furthermore, we have developed a custom assembler to translate assembly files into the instruction ROM for our bespoke instruction set. This set includes: vector loading from memory, vector storage to memory, row-wise addition, row-wise subtraction, row-wise multiplication, row-wise division, row-wise exponential function, row-wise Sigmoid function, normalization via Root Mean Square, and MatMul-free.

Table S10. Performance of NSLLM on FPGA implementation. NSLLM shows a total power consumption of 27.35 W with dynamic power of 13.849 W, inference memory usage of 946 MiB, and an inference throughput of 126.7 tokens/s, while NSLLM* (with spiking sparsity strategy) improves inference throughput to 161.8 tokens/s.

Architecture	Bits	Spike -driven	Time Step	Params (B)	Flops↓ (G)	Winogrande	ARC-E	ARC-C	HeadQA	OpenBookQA	PIQA	Avg↑
RWKV-4	32-32	✗	N/A	1.5	1445.47	55.17	53.32	29.86	27.61	34.40	71.44	45.30
**NSLLM (Ours)**	**1.5-1**	✓	**255**	**1.5**	**3566.84**	**51.07**	**44.36**	**28.84**	**25.42**	**30.00**	**66.76**	**41.07**
**NSLLM* (Ours)**	**1.5-1**	✓	**255**	**1.5**	**3001.69**	**50.51**	**43.56**	**23.98**	**26.26**	**30.20**	**64.64**	**39.86**

Finally, in Table S11, we provide a qualitative analysis of the energy efficiency of NSLLM (AMD / Xilinx Versal VCK190 FPGA) compared to other customized LLM hardware, including FlightLLM[62] (AMD / Xilinx Alveo U280 FPGA, AMD / Xilinx Versal VHK158 FPGA), EdgeLLM[63] (CPU-AMD / Xilinx VCU128 FPGA, CPU-AMD / Xilinx Alveo U280 FPGA), and DuoQ[64] (Alveo U280 FPGA). Since the number of NVLink links does not affect the typical latency-sensitive edge scenario (batch size = 1), we treat NVIDIA A800 as equivalent to NVIDIA A100 as the baseline. Compared to other non-spike-driven customized LLM hardware, such as FlightLLM, EdgeLLM, and DuoQ, which customize LLMs on FPGA using quantization and structural/non-structural sparsity techniques (network-level optimization), NSLLM takes a neuroscience perspective by simulating the sparse computation and event-driven mechanisms of biological systems. It uses spike encoding to reduce LLM energy consumption (encoding-level optimization) while enhancing the system's interpretability through neural dynamics and information processing. Our sparse addition and spike-driven matmul-free LLM design fully leverages biological sparsity, which is key to breaking through energy efficiency bottlenecks. NSLLM achieves significant improvements in power efficiency, memory usage, and inference throughput, with **19.8**×, **21.3**×, and **2.2**× increases, respectively, compared to the A800 GPU, thanks to its hardware-software co-design. At the same time, NSLLM maintains impressive performance

on LLM tasks, a result of our design that uses hybrid time windows and directed sparse quantization, maximizing spike sparsity while preserving spike representation capability. NSLLM, aimed at more adaptable and sustainable intelligent systems, provides a fresh perspective for the development of AGI (Artificial General Intelligence). It explores the integration of biological efficiency, interpretability, and computational power, offering valuable insights for the design of the next generation of neuromorphic chips.

Table S11. NSLLM vs other customized LLM hardware implementations qualitative efficiency comparison. The 'times' values are relative to NVIDIA A100/A800. A800 is a variant of A100, differing only in NVLink links, while other parameters remain the same, so we treat them as equivalent baselines.

Architecture	Spike	Hardware	Power Efficiency		Memory Usage		Inference Throughput	
	-driven	Platform	times	W	times	MiB	times	tokens/s
FlightLLM	✗	Alveo U280 FPGA	4.5×	45	N/A	N/A	N/A	55
		Versal VHK158 FPGA	N/A	155	N/A	N/A	1.2×	92.5
EdgeLLM	✗	CPU-VCU128 FPGA	7.55×	56.8	N/A	N/A	1.9×	N/A
		Alveo U280 FPGA	N/A	155	N/A	N/A	N/A	75
DuoQ	✗	Alveo U280 FPGA	4.8×	N/A	N/A	N/A	N/A	N/A
**NSLLM* (Our)**	✓	Versal VCK190 FPGA	**19.8**×	27.35	↓**21.3**×	946	**2.2**×	161.8

Supplementary Note 14: Neural dynamics and information analysis method

For given spike sequences of neuron α, $\mathbf{s}_\alpha[t,d], d = \{1,2,...,D\}, t = \{1,2,...,t_M\}$, they can be regarded as the outcome of a probabilistic firing model. Thus we have the probability density function (PDF) $p_\alpha(\theta)$ of the spiking activity of neuronal encoder α as follows:

$$p_\alpha(\theta) = \frac{1}{t_M}\sum_{t=1}^{t_M}\delta(\sum_{d=1}^{D}\mathbf{s}_\alpha[t,d]-\theta) \tag{32}$$

where $\delta(\cdot)$ denotes the Kronecker delta function. By converting the spike sequence into a probability distribution, we can derive the information entropy metrics of the corresponding spike sequence to evaluate the temporal dynamics of neuronal encoders in NSLLMs.

The mean Shannon entropy $H(X)$ of each layer X is calculated to evaluate information richness and feature diversity in our models. Increased Shannon entropy in brain networks has been associated with altered states of consciousness of the corresponding functional areas. For instance, Shannon entropy can be applied as a model-free method to detect brain activation from fMRI time series[65,66]. The mean Shannon entropy is derived as:

$$\begin{aligned} H(X) &= \frac{1}{n_X}\sum_{i=1}^{n_X}H(X_i) \\ &= -\frac{1}{n_X}\sum_{i=1}^{n_X}\int p_{X_i}(\theta)\ln p_{X_i}(\theta)\mathrm{d}\theta \end{aligned} \tag{33}$$

where $p_{X_i}(\theta)$ denotes the marginal probability density function of encoder θ of neuron X_i from layer X, and n_X is the number of neurons in layer X. A higher mean Shannon entropy $H(X)$ signifies the higher encoding capacity for neurons in layer X.

To assess the representational capability of NSLLM, we can calculate the mean normalized mutual information (MI) $\widetilde{I}(X,Y)$ (to normalize the influence of Shannon entropy on mutual information) between the initial input layer X and intermediate hidden layers Y. Mutual information measures the amount of information shared between neural stimuli (initial/input layer) and responses (hidden layer), providing insights into how effectively information is transmitted within neural systems. For instance, mutual information is used to detect spurious long-range temporal dependencies and structural dependencies in resting-state neuroimaging data[67,68]. The mean normalized MI between two layers X and Y is calculated as follows:

$$\begin{aligned} \widetilde{I}(X,Y) &= \frac{2}{H(X)+H(Y)}I(X,Y) \\ &= \frac{2}{H(X)+H(Y)}\frac{1}{n_X n_Y}\sum_{i=1}^{n_X}\sum_{j=1}^{n_Y}\int\int p_{X_i,Y_j}(\theta,\psi)\ln\left(\frac{p_{X_i,Y_j}(\theta,\psi)}{p_{X_i}(\theta)p_{Y_j}(\psi)}\right)\mathrm{d}\theta\mathrm{d}\psi \end{aligned} \tag{34}$$

$$p_{X_i,Y_j}(\theta,\psi) = \frac{1}{t_M}\sum_{t=1}^{t_M}\delta(\sum_{d=1}^{D}\mathbf{s}_{X_i}[t,d]-\theta)\delta(\sum_{d=1}^{D}\mathbf{s}_{Y_j}[t,d]-\psi) \tag{35}$$

where $p_{X_i,Y_j}(\theta,\psi)$ represents the joint probability density function (PDF) of encoder θ of neuron X_i from layer X and encoder ψ of neuron Y_j from layer Y; $p_{X_i}(\theta)$ and $p_{Y_j}(\psi)$ denote the marginal PDFs of their respective distributions. A higher mean normalized MI $\widetilde{I}(X,Y)$ indicates that layer Y more effectively captures and encodes the information presented in layer X.

To examine the dynamic properties of the NSLLM, we employed Kolmogorov-Sinai entropy (KS entropy)[24,48]), which quantifies the neural dynamics complexity or dynamic randomness of the encoding neurons within different layers. KS entropy can be used to assess the complexity of neural dynamics, with higher values indicating more chaotic and unpredictable behavior, which may correlate with certain cognitive states. For instance, KS entropy, along with other nonlinear measures, is applied to quantify the unpredictability in EEG signals, demonstrating its effectiveness in distinguishing between different emotional states and supporting the presence of chaotic dynamics in human brain activity[69–71]. The KS entropy H_{KS} of a neural activity sequence $\mathcal{S}$ from time t to time $t+\tau$ is defined as

$$H_{KS}(\mathcal{S},t,t+\tau) = -\frac{1}{\tau}\sum_{r}\sum_{r'>r}P(r\mid\mathcal{S},0,t)W_{rr'}(\mathcal{S},t,t+\tau)\ln W_{rr'}(\mathcal{S},t,t+\tau) \tag{36}$$

where t is the reference time, τ is the length of the time interval, r and r' represent the total spike times within a given period, $P(r \mid \mathcal{S}, 0, t)$ is the probability of observing r spikes from time 0 to t given the activity sequence $\mathcal{S}$, and $W_{rr'}(\mathcal{S}, t, t+\tau)$ denotes the transition probability of spike counts from r to r' during the interval $[t, t+\tau]$ with $\mathcal{S}$. We assumed that the neural activity of each encoding neuron from different layers of the LLMs adheres to an inhomogeneous Poisson process, so we can theoretically derive that

$$P(r \mid \mathcal{S}, t, t+\tau) = \frac{\Lambda^r(t, t+\tau)}{r!} e^{-\Lambda(t,t+\tau)} \tag{37}$$

$$W_{rr'}(\mathcal{S}, t, t+\tau) = P(r \mid \mathcal{S}, 0, t) P(r' - r \mid \mathcal{S}, t, t+\tau) \tag{38}$$

where $\Lambda(t, t+\tau) = \int_t^{t+\tau} \lambda(u) \mathrm{d}u$ is the integral of the time-variant intensity $\lambda(t)$ of the inhomogeneous Poisson process. To compute the KS entropy in Eq. 36, we took the maximum likelihood estimation (MLE) of $\Lambda(t, t+\tau)$ as follows:

$$\widehat{\Lambda}(t, t+\tau) = \sum_{u=t}^{t+\tau} \widehat{r}_u = \sum_{u=t}^{t+\tau} \sum_{d=1}^{D} \mathbf{s}[u, d] \tag{39}$$

where $\widehat{r}_t = \sum_{d=1}^{D} \mathbf{s}[t, d]$ denotes the observed output (sum of the spike sequence) at time step t. A higher KS entropy indicates a stronger capability of the system to process diverse information.

Supplementary Note 15: Extend neural dynamical analysis of NSLLM outcome

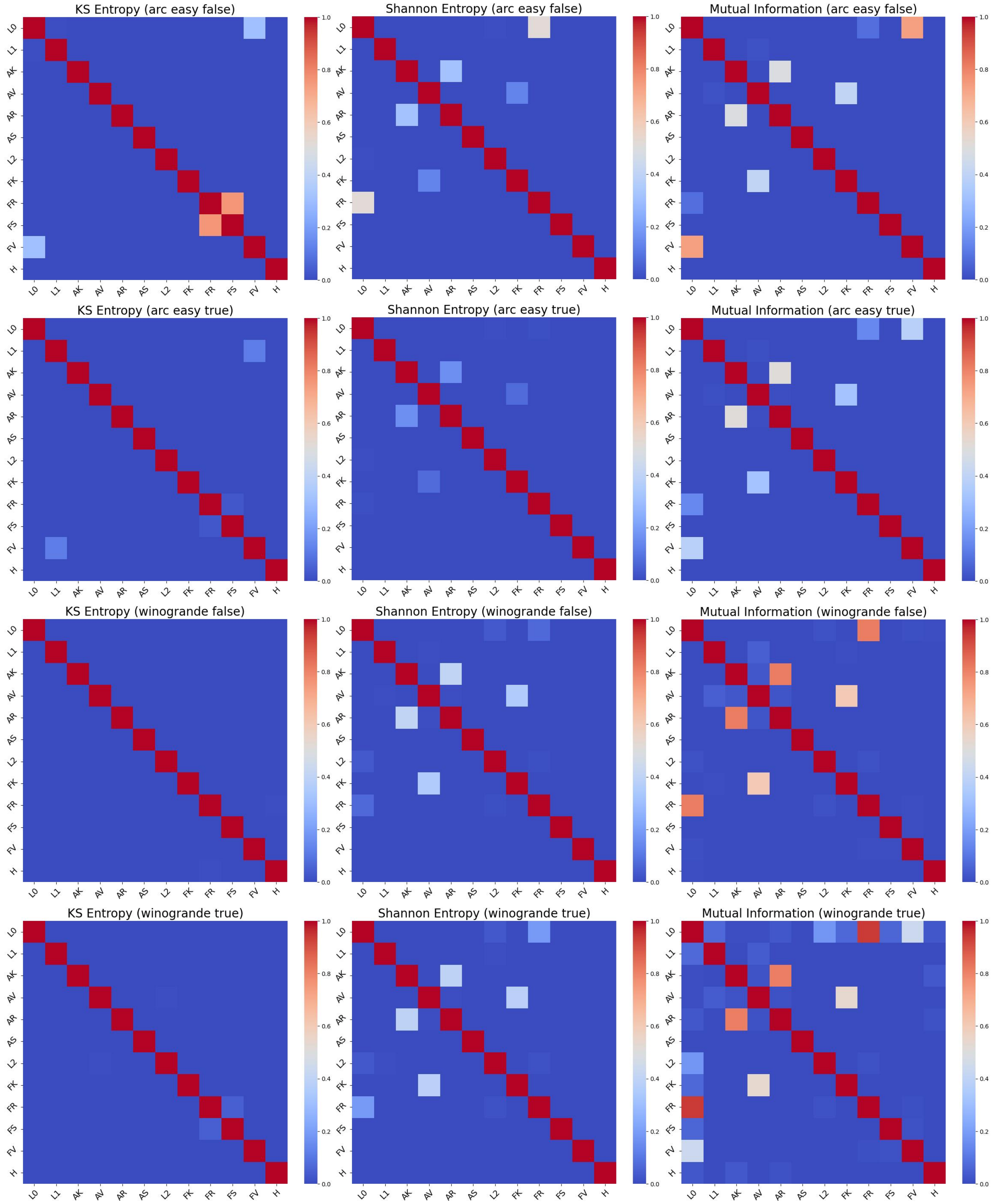

Figure S10. The significance test (t-test, two-tailed) p-values of statistic results in Figs. 6b-d. KS entropy (left column, extended data figure for Fig. 6b), Shannon entropy (middle column, extended data figure for Fig. 6c), mutual information (right column, extended data figure for Fig. 6d).

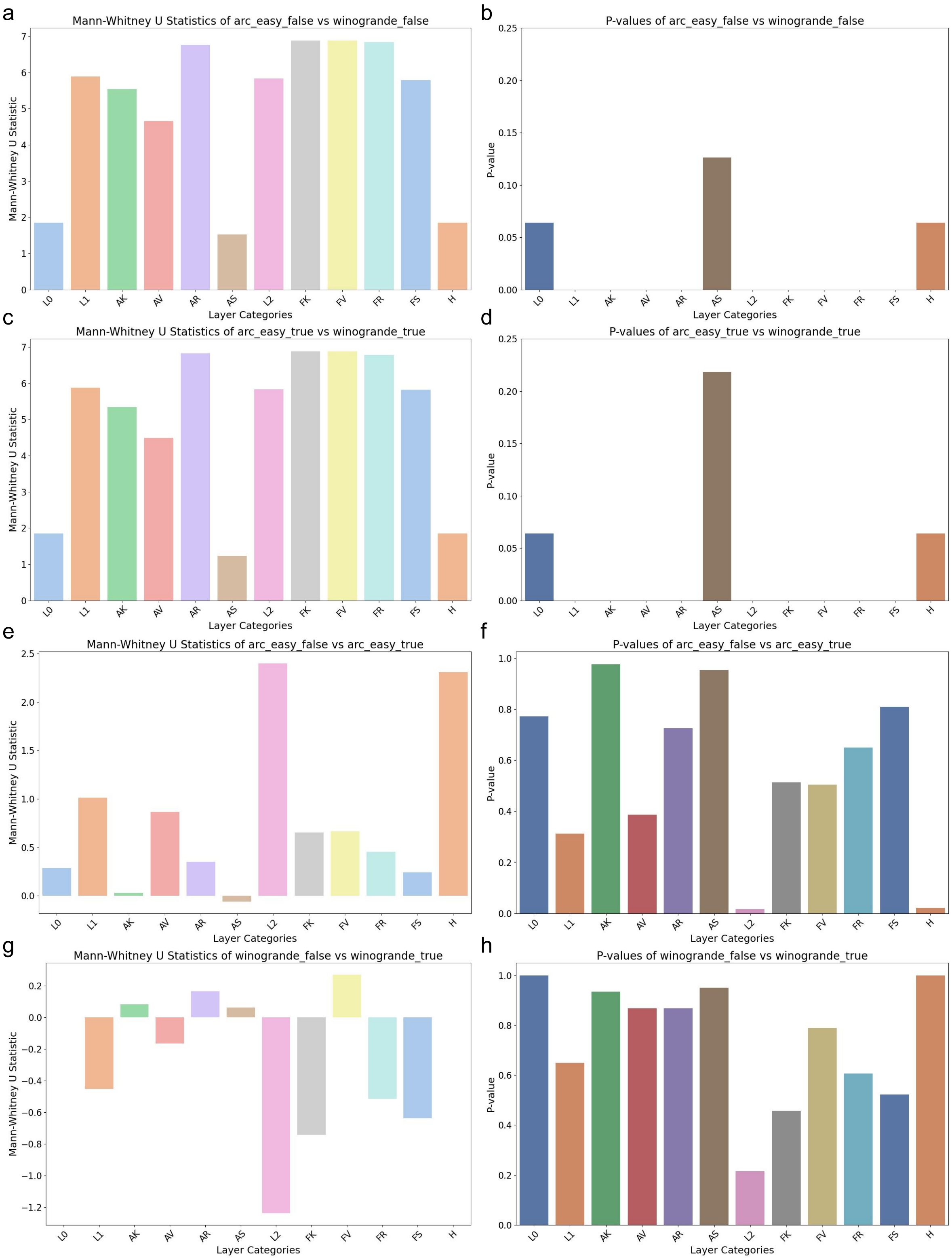

Figure S11. The extended result figure of Figs. 7c-f. The Mann-Whitney U statistics results show that the NSLLM framework can effectively discriminate ambiguous/unambiguous datasets by mutual information (normalized), as shown in subfigures a-d. However, it performs similarly on tasks on the correct/incorrect datasets, as shown in subfigures e-h.

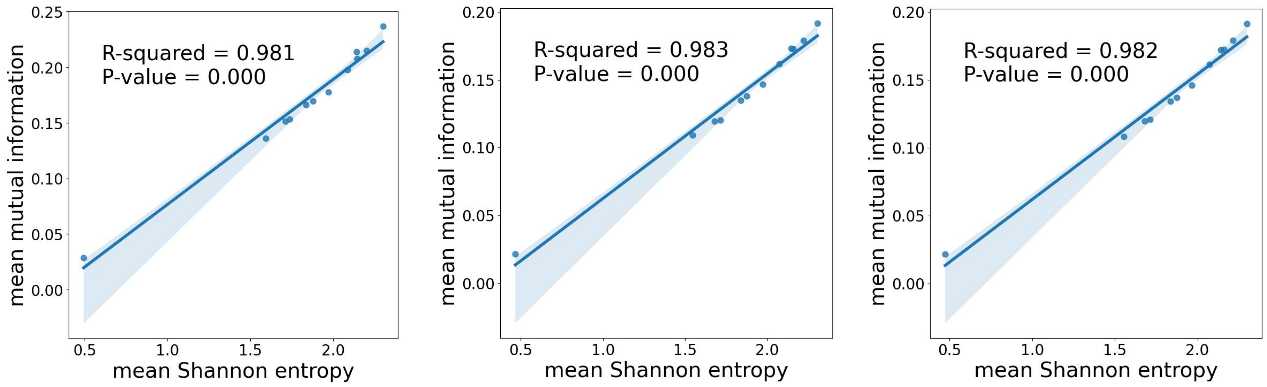

Figure S12. The extended result figure of Fig. 7a. The Pearson correlation analysis results of the dataset 'arc easy false' (left), 'winogrande true' (middle), 'winogrande false' (right).

Supplementary Note 16: Tasks and experimental setup

To comprehensively evaluate the performance of NSLLM, we selected a variety of challenging benchmark datasets that cover key areas including common-sense reasoning, scientific knowledge, background knowledge application, physical interaction understanding, and creative thinking, as follows: Winogrande[72] is a dataset designed to evaluate a language model's ability to solve problems that require common-sense reasoning. It contains 44,000 questions, each with two answer choices, one of which is correct. The questions require understanding relationships between entities and the semantic roles in the given context. ARC[73] is a dataset for assessing AI systems' ability to solve scientific questions, consisting of nearly 8,000 questions. It is divided into two subsets: ARC Easy and ARC Challenge. ARC Easy includes questions answerable by simple information retrieval, while ARC Challenge requires deeper understanding and reasoning. OpenBookQA[74] is a dataset that evaluates AI systems' ability to answer questions that require background knowledge. It includes 6,000 questions, each associated with a list of options and a relevant scientific fact. OpenBookQA requires the system to combine the question with the fact and use reasoning to select the correct answer. PIQA[75] is a dataset designed to evaluate an AI system's ability to understand the semantics of physical interactions and events in the world. It contains nearly 21,000 questions, each with two answer choices, where one is correct. The questions are crafted to assess understanding of causal relationships and object properties in the physical world. HeadQA[76] is a dataset that evaluates an AI system's ability to answer questions requiring brainstorming and creative thinking. It contains 2,680 questions, each with multiple answer choices, where one is correct. The goal is to assess the AI's ability to generate novel and creative responses rather than merely retrieving information. BoolQ[77] is a dataset designed to evaluate AI systems' ability to handle binary fact-based questions. It contains approximately 16,000 questions, each requiring a "yes" or "no" answer based on the provided context. The dataset aims to assess models' ability to make judgments after understanding long-form textual inputs. HellaSwag[73] is a dataset for evaluating AI systems on common-sense reasoning and event completion tasks. It includes around 70,000 questions, each requiring the selection of the most plausible event continuation from four given choices. The HellaSwag task is particularly challenging and designed to test models' reasoning and contextual understanding abilities.

Meanwhile, we adopted the following experimental setup for our models: We used the FuseAdam optimizer to accelerate the training process, with a first moment estimate of 0.9, a second moment estimate of 0.99, an initial learning rate of 8×10^{-4}, and step-based decay. The context length is set to 1024. These configurations were applied to all our ablation experiments and the final models presented. We trained our models on A800 GPUs using commonly used datasets, with a relatively small batch size and few training epochs. Once trained, the models were deployed on FPGAs for inference.
